# *In Vivo* Gene Expression Profiling of Staphylococcus aureus during Infection Informs Design of Stemless Leukocidins LukE and -D as Detoxified Vaccine Candidates

**DOI:** 10.1128/spectrum.02574-22

**Published:** 2023-01-23

**Authors:** Andreas F. Haag, Lassi Liljeroos, Paolo Donato, Clarissa Pozzi, Tarcisio Brignoli, Matthew J. Bottomley, Fabio Bagnoli, Isabel Delany

**Affiliations:** a GSK, Siena, Italy; b School of Medicine, University of St. Andrews, St. Andrews, United Kingdom; c Roche Oy, Espoo, Finland; d School of Cellular and Molecular Medicine, University of Bristol, Bristol, United Kingdom; University of Calgary

**Keywords:** *in vivo* gene expression, high-throughput qRT-PCR, *Staphylococcus aureus*, *Staphylococcus aureus* vaccine, leukocidins

## Abstract

Staphylococcus aureus is a clinically important bacterial pathogen that has become resistant to treatment with most routinely used antibiotics. Alternative strategies, such as vaccination and phage therapy, are therefore actively being investigated to prevent or combat staphylococcal infections. Vaccination requires that vaccine targets are expressed at sufficient quantities during infection so that they can be targeted by the host’s immune system. While our knowledge of *in vitro* expression levels of putative vaccine candidates is comprehensive, crucial *in vivo* expression data are scarce and promising vaccine candidates during *in vitro* assessment often prove ineffective in preventing S. aureus infection. Here, we show how a newly developed high-throughput quantitative reverse transcription-PCR (qRT-PCR) assay monitoring the expression of 84 staphylococcal genes encoding mostly virulence factors can inform the selection and design of effective vaccine candidates against staphylococcal infections. We show that this assay can accurately quantify mRNA expression levels of these genes in several host organs relying only on very limited amounts of bacterial mRNA in each sample. We selected two highly expressed genes, *lukE* and *lukD*, encoding pore-forming leukotoxins, to inform the design of detoxified recombinant proteins and showed that immunization with recombinant genetically detoxified LukED antigens conferred protection against staphylococcal skin infection in mice. Consequently, knowledge of *in vivo*-expressed virulence determinants can be successfully deployed to identify and select promising candidates for optimized design of effective vaccine antigens against S. aureus. Notably, this approach should be broadly applicable to numerous other pathogens.

**IMPORTANCE** Vaccination is an attractive strategy for preventing bacterial infections in an age of increased antimicrobial resistance. However, vaccine development frequently suffers significant setbacks when candidate antigens that show promising results in *in vitro* experimentation fail to protect from disease. An alluring strategy is to focus resources on developing bacterial virulence factors that are expressed during disease establishment or maintenance and are critical for bacterial in-host survival as vaccine targets. While expression profiles of many virulence factors have been characterized in detail *in vitro*, our knowledge of their *in vivo* expression profiles is still scarce. Here, using a high-throughput qRT-PCR approach, we identified two highly expressed leukotoxins in a murine infection model and showed that genetically detoxified derivatives of these elicited a protective immune response in a murine skin infection model. Therefore, *in vivo* gene expression can inform the selection of promising candidates for the design of effective vaccine antigens.

## INTRODUCTION

Staphylococcus aureus is a remarkably versatile pathogen that can be found as a commensal colonizing the skin and nares of up to 30% of the population (1). Regardless of its commensal lifestyle, S. aureus has evolved into one of the most prevalent human pathogens in both hospital- and community-acquired infections, and the threat of S. aureus resistant to antimicrobials is high (2–4). The pathogen can cause a plethora of disease manifestations ranging from skin and soft tissue to invasive infections (5, 6). Virulence factor expression is controlled by an intricate regulatory network that executes differential gene expression in responses to multiple signals from the host environment and the bacterial population (7–10).

S. aureus can acquire new virulence traits through horizontal gene transfer, and single transfer events can render a previously nonpathogenic strain multidrug resistant and/or hypervirulent (11–14). Alternative strategies for the prevention of staphylococcal infection/colonization rather than treatment of the disease would be the preferred choice for combating the dissemination of increasingly virulent and resistant strains (15). Indeed, the WHO rates S. aureus among the top 12 bacterial pathogens requiring urgent attention by the research community for the development of new treatment and prevention strategies (2). Vaccination against bacterial pathogens has been very successful in limiting and even eliminating specific diseases, is cost efficient (16), and could provide a viable strategy for the prevention of staphylococcal infections. No correlate of protection is currently known for staphylococcal infection, rendering vaccine development an arduous task (6). Indeed, to date S. aureus has resisted efforts to develop an effective vaccine able to elicit protective immunity in humans (17, 18).

One of the hurdles in developing an efficacious vaccine against S. aureus infections is the number and complexity of bacterial immune evasion factors expressed (19), highlighting the remarkable adaptation of S. aureus to its host. In fact, as S. aureus normally colonizes its host asymptomatically, these sophisticated mechanisms are highly successful in dampening the host immune defenses and preventing them from controlling the pathogen (20, 21). Moreover, it is not fully understood which of the known virulence determinants of S. aureus are critical during infection, as different combinations and regulation of virulence determinants in different lineages can have a profound impact on bacterial virulence (10, 22). Few studies have measured differential gene expression during animal or human infection (23–30). Studying these variations can give important insights into which antigens the pathogen expresses in the host, how it adapts to the host environment, and how this response translates into different types of disease. In the current age of highly sophisticated transcriptomic technologies, discovery of promising vaccine candidates needs to be further guided by expression data of antigens from *in vivo* models or, better still, from patients during acute or chronic infection with the pathogen. Gene expression levels in general can be regarded as a good correlate for protein expression and can therefore be used to infer whether a protein or virulence factor is expressed. Such data can provide a more informed approach for the selection of vaccine antigens involved in key pathogenic processes.

The aim of this study was to develop a high-throughput assay for measuring expression of Staphylococcus virulence genes to facilitate the selection of promising vaccine antigens that are expressed during infection. To this end, we designed and optimized an array of quantitative reverse transcription-PCR (qRT-PCR) assays in conjunction with a high-throughput (HT) microfluidics system (31) to simultaneously quantify the expression levels of more than 80 staphylococcal virulence factors and regulatory genes with minute quantities of bacterial RNA. The aim was to enable measurement of the expression levels of S. aureus virulence genes from a large set of host-derived samples during infection within an individual host rather than within pooled samples, enabling us to also address interhost expression variability. Comparison of *in vivo* and *in vitro* expression data revealed the genes with significant upregulation of expression under infection conditions. We further selected two of these genes, encoding the leukocidins LukE and LukD, to test their efficacy as vaccine antigens by designing recombinant antigens and testing their ability to induce protective immunity against S. aureus in a mouse skin infection model.

## RESULTS

### Setup of a high-throughput linear qRT-PCR assay for staphylococcal virulence gene expression profiling.

**FIG 1:**
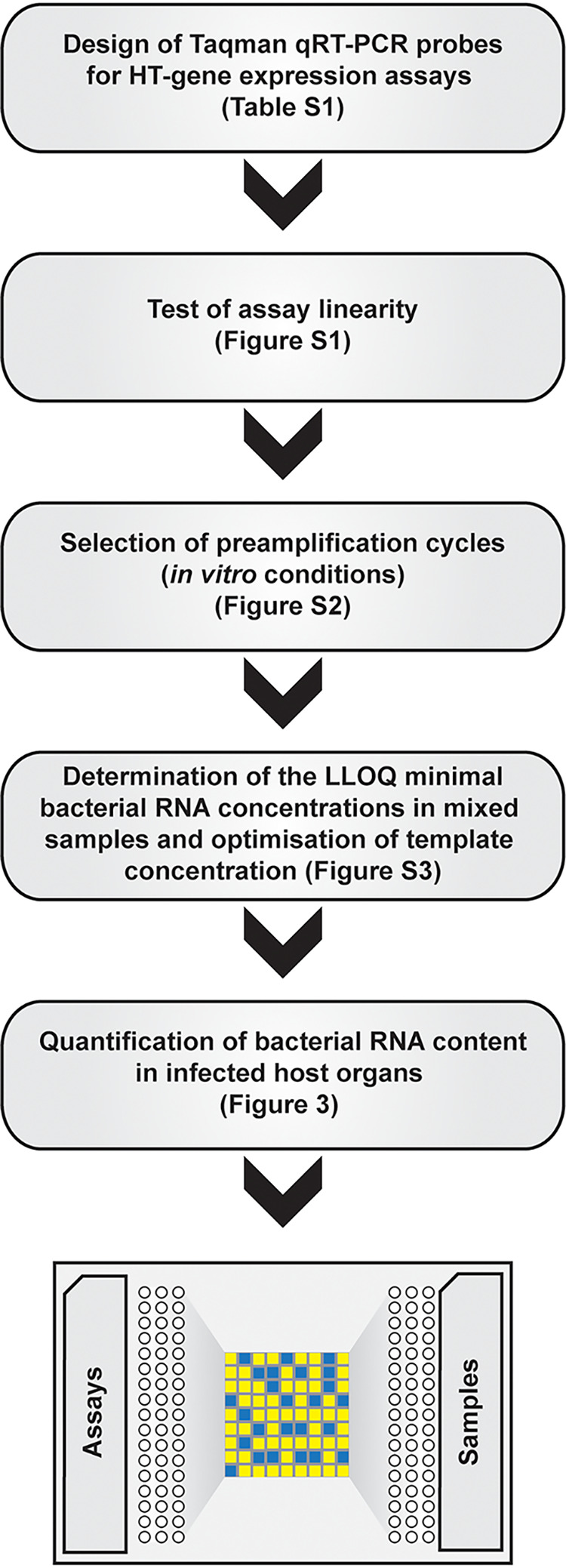
Setup of HT-qRT-PCR assay for *in vivo* expression analysis.

S. aureus can express many different and sometimes redundant virulence factors. To develop a technology to monitor expression of a large set of relevant genes in a high-throughput manner, we set up a simple workflow (Fig. 1). We selected a set of virulence genes involved in various processes during staphylococcal infection (see Table S1 in the supplemental material). Our aim was to cover many different stages of the S. aureus infection process, such as adhesion, invasion, and immune evasion. For this purpose, we included adherence factors, such as MSCRAMMs (32), proteases, toxins, and other immune evasion factors in the panel monitored in our high-throughput screen. To determine the limit of similarity of genes that can be identified specifically, we also included a family of highly similar S. aureus genes that encode conserved staphylococcal antigens (Csa proteins) and which can elicit cross-protective immunity (33). Included also in the list are probes against antigens contained by former or current candidate vaccine formulations: IsdB, capsule type 5 and 8 biosynthetic genes (encoding CP5 and CP8), ClfA and MntA (the vaccine antigen is MntC, which is encoded by the same operon), EsxA, EsxB, FhuD2, Hla, Csa1A, and SpA (reviewed in references 34 to 37). To cover a broad range of staphylococcal isolates, we compared variants of each gene from the five different staphylococcal strains spanning a range of clonal complexes (Newman, USA400 MW2, USA300 FPR3757, and USA100 Mu50 and N315) (Table S2) and designed specific probes (Fig. 1) for conserved regions within each gene (Table S1) for use in a high-throughput microfluidics qRT-PCR (HT-qRT-PCR) system.

Quantification of transcript levels using the HT-qRT-PCR system relies on the preamplification of a cDNA sample prior to assessment by quantitative PCR (qPCR). Therefore, we first assessed the linearity of the qRT-PCR results using different preamplification cycles with the designed probe assays and 25 ng of cDNA prepared from exponentially grown S. aureus strain Newman. We observed that most of our assays performed well, exhibiting a linearity with a slope of −1 throughout the range of preamplification cycles tested (Fig. S1). Values outside the linear spectrum were mostly found at either the lowest or highest number of preamplification cycles and were likely caused either by the limited amount of template in the assay or by saturating the qRT-PCR with too much template, respectively. Assays differing from a slope of −1 by more than 20% or showing a regression coefficient of less than 0.90 were either redesigned (3 assays with *hlgABC*) or excluded from the final panel of assays (Table S1).

Many genes are regulated in a growth-phase-dependent manner and can vary in their steady-state levels, reaching maximal or minimal expression levels at early or late time points accordingly. We investigated whether relative gene expression was altered when using different numbers of preamplification cycles throughout the time course (optical density at 600 nm [OD_600_] values of ~0.5, 2.0, 4.0, 8.0, and 12.0) of an *in vitro* culture by normalizing the expression values of each gene to the expression values of the housekeeping gene *gyrB* and calculating the relative gene expression levels across the time course (Fig. S2). While we observed an increased variability for certain subsets of genes using either fewer or more preamplification cycles, this did not affect the overall expression patterns for each of the genes when looking at relative expression values (Fig. S2B to F). Using principal-component analysis to determine the similarity of different expression data sets, we observed that samples taken at the same growth phase clustered together irrespective of the number of preamplification cycles used (Fig. S2A), indicating that different preamplification cycles did not significantly alter the overall gene expression profiles determined. However, we observed that a small number of assays performed less well at higher preamplification cycles. Consequently, we selected 14 preamplification cycles as the approach for accurate quantification of relative transcript levels, applicable for both lowly and highly expressed genes.

### S. aureus virulence genes show distinct expression kinetics *in vitro*.

**FIG 2:**
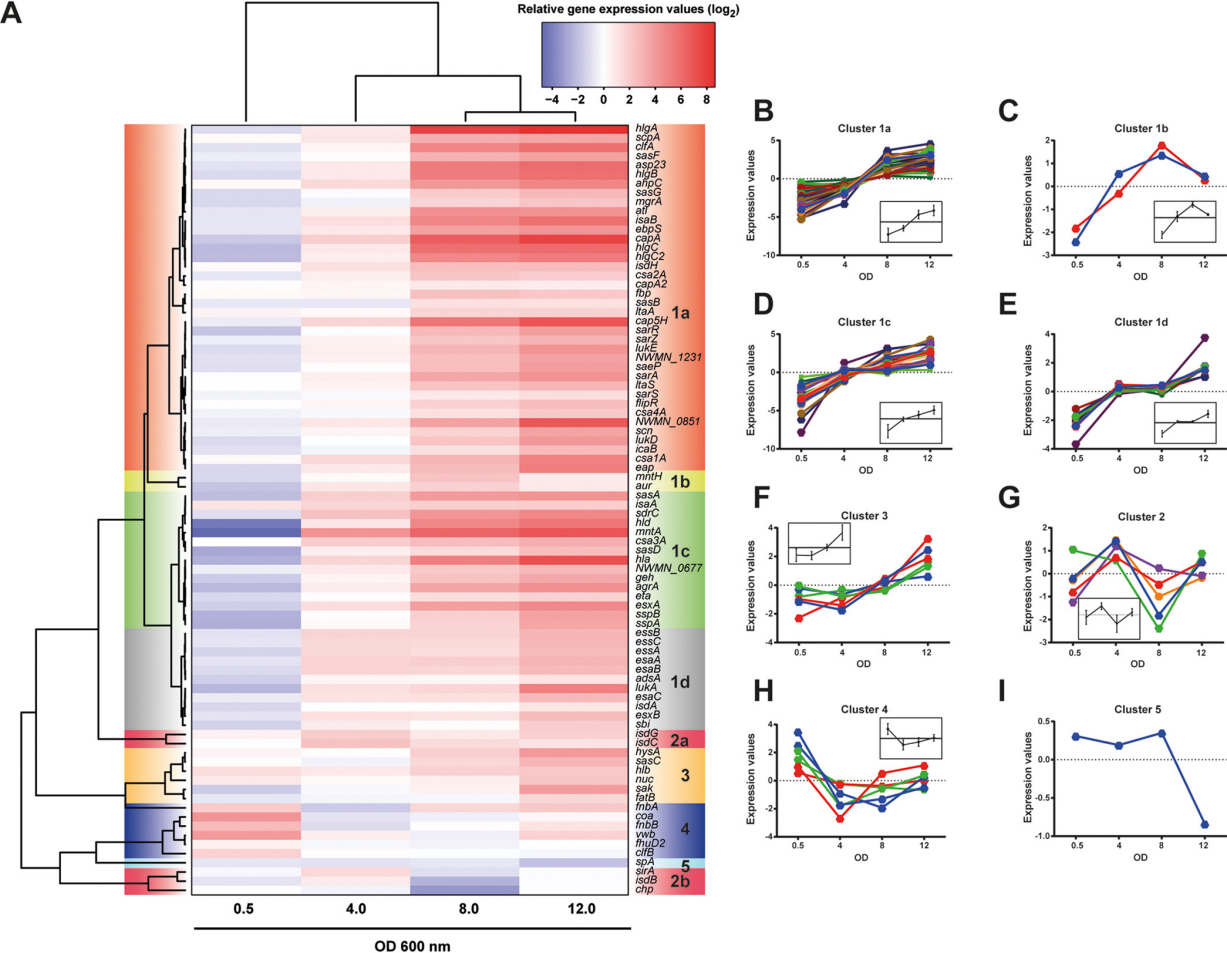
Characterization of gene expression levels throughout *in vitro* growth. S. aureus strain Newman was grown *in vitro*, and samples for RNA extraction were taken at the defined ODs. The obtained *C_T_* values of the different samples were normalized to the levels of gyrase B (*gyrB*). (A) Heat map and hierarchical clustering of relative expression levels to exponential-phase cultures (OD_600_ of 2). (B to I) Expression profiles of target genes during *in vitro* growth using K-medoids-clustering analysis. Values calculated from this analysis were used to graph the data set reflecting the clusters from panel A. Inlays represent the overall trend within each cluster (B to I).

Differences in gene expression profiles for all genes throughout the bacterial growth were calculated relative to an OD_600_ of ~2.0 (mid-exponential phase [the OD later used as the inoculum in infection experiments]), and hierarchical clustering was performed using Euclidian distance and complete linkage, giving rise to the clusters observed in the heat map (Fig. 2) and as shown in Table S3. As expected, the expression of the majority of genes analyzed (72/84) increased with bacterial cell density (Fig. 2A, clusters 1a to -d, 2a, and 3), with the main differences observed in the timing and degree of activation of gene expression (Fig. 2B to F). All known regulators of gene expression assessed in our assay (*sarA*, -*R*, -*S*, and -*Z*, *mgrA*, *agrA*, and *saeP*) increased expression with increasing cell density, indicating that these might play an active role in bacterial adaptation to higher cell densities. Correspondingly, genes under the control of these regulators were more highly expressed. For example, expression of the leukocidin genes *lukED*, *lukAB*, and *hlgABC* (Fig. 2, cluster 1a) has previously been shown to be activated by the SaeRS two-component signal transduction system (TCS) (38), while genes known to be activated by the Agr quorum sensing TCS (i.e., capsule genes *hla* and *hld*) (Fig. 2, cluster 1c) were expressed during the stationary phase (9). Conversely, expression of *spA* (Fig. 2, cluster 5), encoding the immune evasion staphylococcal protein A, remained constant during exponential growth but was downregulated in stationary-phase cultures (Fig. 2I) similarly to genes in cluster 2b. Several genes attributed to bacterial adhesion (*fnbA* and -*B*, *coa*, *vwb*, and *clfB*) (Fig. 2, cluster 4) as well as the iron-responsive *fhuD2* gene were expressed during exponential growth and downregulated in the stationary phase (Fig. 2H). While *clfB*, encoding clumping factor B, followed this pattern, *clfA* (in cluster 1a), encoding clumping factor A, was induced in stationary-phase cultures relative to exponential-phase bacteria (Fig. 2B). Expression of the staphylococcal type VII secretion system was also induced with progression of bacterial growth in the culture (Fig. 2A, clusters 1c and -d). Overall, these results show that our qRT-PCR assay was able to reveal distinct S. aureus gene expression profiles for the tested bacterial virulence factors/regulators *in vitro*.

### Relative expression of bacterial virulence genes can be accurately measured in mixed RNA samples with minute bacterial RNA quantities.

RNA isolated during bacterial infection of host tissues often consists mainly of host RNA and contains only a small fraction of bacterial RNA. In order to determine the minimum bacterial RNA load required to accurately quantify relative gene expression levels (lower limit of quantification [LLOQ]), we prepared mock mixed RNA samples using total RNA extracted from uninfected mouse kidneys mixed with defined quantities of total bacterial RNA. We generated 2 sets of mock mixed RNAs with ratios ranging from 1:1 to 1:1,000,000 (50% to 0.000001%) using bacterial RNA from exponential-phase (OD_600_ = 0.5) and stationary-phase (OD_600_ = 12) cultures of *in vitro*-grown S. aureus to allow for the maximum range of expression for each gene, while pure bacterial cDNA served as reference for the highest detectable signal and to calibrate the relative expression calculations. Only 14 preamplification cycles resulted in expression data comparable to those of pure bacterial RNA/cDNA (data not shown), while more preamplification cycles did not result in better quantitative determination of expression values. This suggested that the type of sample (i.e., mixed versus pure) highly influenced its amenability to preamplification and that accurate relative gene expression quantification could be achieved in mixed bacterial and host RNA samples when performing up to 14 cycles of preamplification. Furthermore, we observed that a ratio of 1:100 or 1:1,000 (1 to 0.1%) of bacterial to host RNA was sufficient for accurate quantification of target genes expressed at intermediate levels when using 25 ng of cDNA starting material (Fig. S3). We tested whether the amount of starting cDNA prior to preamplification could result in better rates of quantification within highly dilute samples. As before, we used the same mixed cDNA samples and subjected 25, 125, and 250 ng of template cDNA to 14 cycles for the preamplification. As expected, threshold cycle (*C_T_*) values decreased with increasing template amounts in the preamplification reaction (data not shown) and increasing the template amount 10-fold (250 ng) enabled the reproducible detection and quantification of relative gene expression values in samples as dilute as 0.01% of bacterial RNA in the starting material. A Spearman’s correlation analysis of all gene expression values (Fig. S3) between mock host-derived samples and pure bacterial cDNA samples showed a high correlation of gene expression patterns between pure bacterial RNA down to 0.01% of initial bacterial RNA content in mixed samples.

### Bacterial RNA content varies considerably among infected host organ extracts.

**FIG 3:**
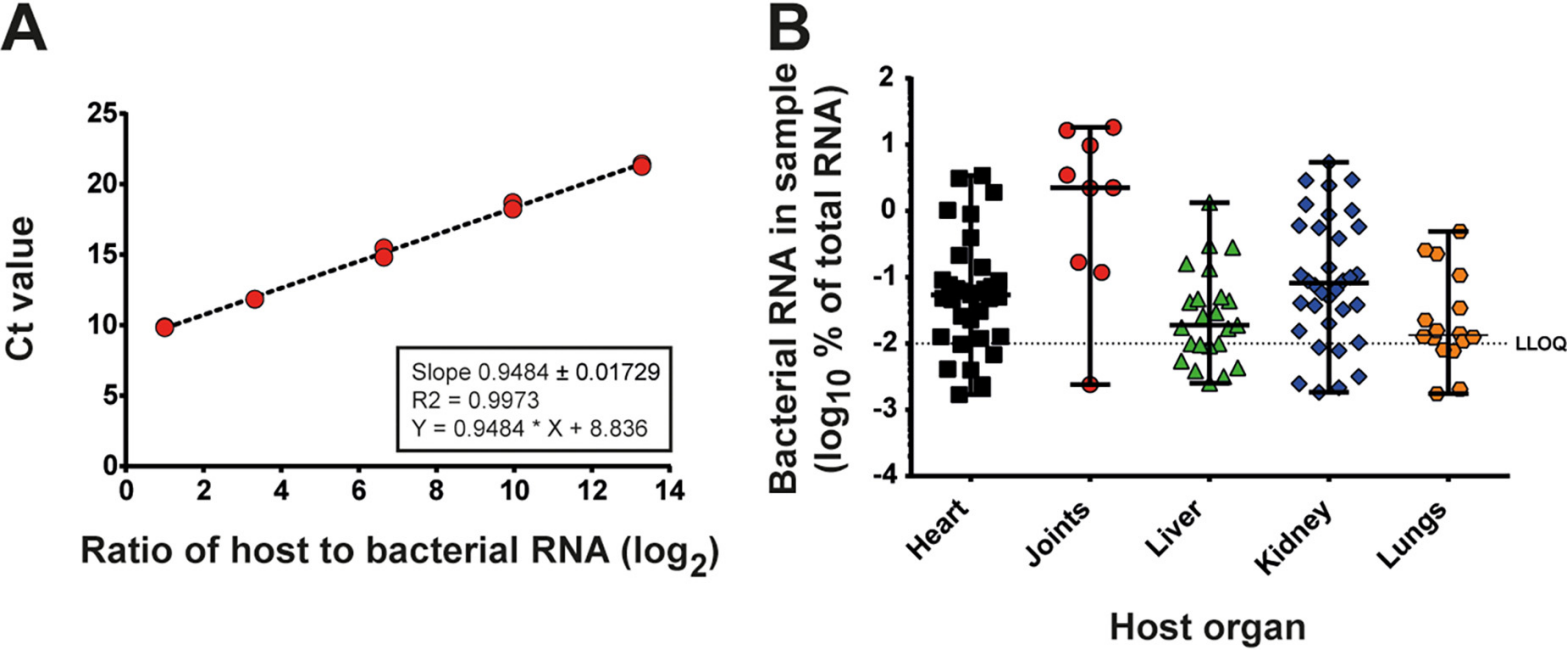
Bacterial RNA content of host organ samples. RNA was extracted from infected host organs, and the relative content of bacterial RNA in the mixed RNA samples was determined by measuring the bacterial 16S rRNA and relating it to a calibration curve of defined ratios of bacterium to host RNA. (A) Calibration curve of defined ratios of bacterial and mouse RNA shown as ratios of host to bacterial RNA. (B) cDNAs were prepared from mouse organs over a period of 14 days postinfection, and the bacterial RNA content was determined using the calibration curve shown in panel A. The lower limit of quantification (LLOQ) identified in the *in vivo* mock experiments (described below) allowing a reliable quantification of relative gene expression levels is indicated.

Having determined the technical limitations of our HT-qRT-PCR system, we assessed the feasibility of applying it to samples extracted from host infections. CD1 mice were intravenously infected with strain Newman, and infected organs were harvested at 2, 4, 7, and 14 days postinfection. We isolated mixed RNA from these infected mouse organs (39) and determined the relative proportion of bacterial RNA in each sample (Fig. 3). This analysis was performed using standard qRT-PCR protocols and probes targeting 16S rRNA against a calibration curve of cDNA generated from various dilutions of pure bacterial RNA mixed with pure mouse RNA (Fig. 3A). We observed that bacterial RNA yields varied widely within and between different host organs (Fig. 3B). Detectable levels of bacterial RNA ranged in general between 0.001 and 10% of total RNA. Hearts and kidneys yielded the highest average percentages of bacterial RNA being ~10 times more concentrated than RNA extracted from infected livers and lungs (Fig. 3B), in line with the bacterial burden in the different organs (39). Given the distribution of bacterial RNA content in host-extracted samples, the majority of samples from heart and lung would therefore be near the lower limit of quantification when using 25 ng of starting material for *in vivo*-derived samples. Consequently, larger amounts of total, mixed RNA were required to ensure the reliable quantification gene expression from these samples.

### *In vivo* expression of bacterial virulence genes is significantly different from that in *in vitro*-grown cultures.

**FIG 4:**
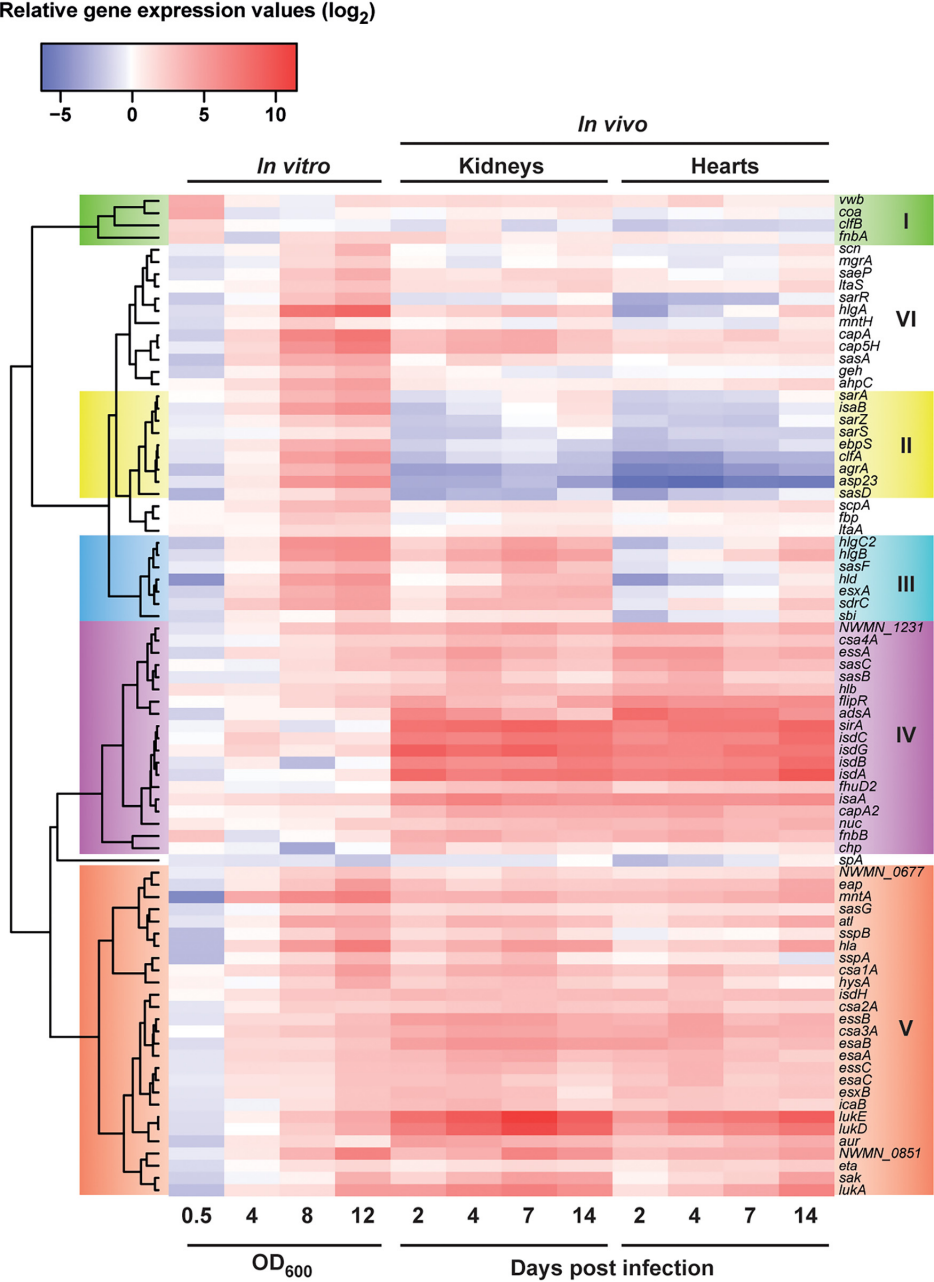
Comparison of levels of *in vitro* and *in vivo* gene expression. Shown is a heat map of virulence factor expression profiles throughout *in vitro* growth or infection of mouse kidneys or hearts. Expression levels were normalized to *gyrB* and are presented relative to the bacterial inoculum used for infection (OD_600_ of 2 in TSB).

**FIG 5:**
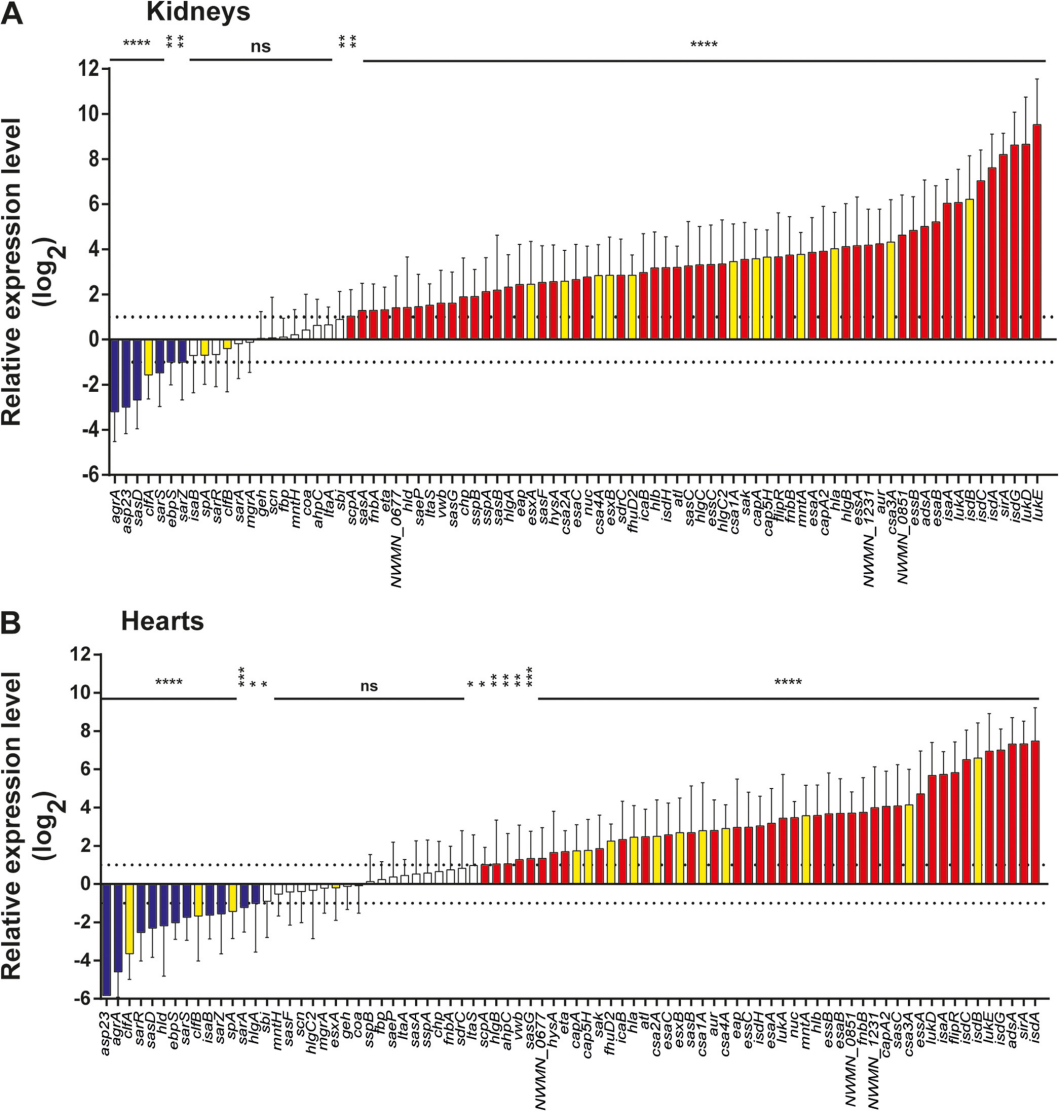
Relative expression levels of all genes *in vivo* in kidney (A) and heart (B) tissue in relation to the inoculum. Current vaccine antigens are highlighted in yellow. Expression levels were normalized to *gyrB* and are presented relative to the bacterial inoculum used for infection (OD_600_ of 2 in TSB) combined for all time points *in vivo* and ordered according to fold change. Error bars show the standard deviation from the mean, and statistical analysis was performed by one-way ANOVA followed by Holm-Sidak’s multiple-comparison test relative to the inoculum. Statistical significance levels are indicated as follows: ns, not significant; *, *P* < 0.05; **, *P* < 0.01; ***, *P* < 0.001; ****, *P* < 0.0001.

To reproducibly determine the relative gene expression levels *in vivo*, we therefore used 250 ng of host-extracted, mixed cDNA samples, while we used 25 ng of pure bacterial cDNA from *in vitro*-grown samples for our HT-qRT-PCR assay. S. aureus cultures used as the inoculum prior to infection (OD_600_ = 2.0) served as the baseline for bacterial gene expression levels, and cDNA prepared from uninfected mouse kidneys served as the control for background and unspecific amplification. No amplification for any of the S. aureus assays was observed in RNA extracted from uninfected mouse kidneys (data not shown). We observed comprehensive overall coverage of quantifiable gene expression in kidney and heart samples (Fig. S4). Overall differential gene expression profiles separated clearly into two discrete groups consisting of either *in vitro* or *in vivo* profiles (Fig. S5). In general, most genes were induced in host-extracted samples relative to the inoculum and genes in clusters IV and V are equally upregulated in both kidney and heart tissues (Fig. 4). Unsurprisingly, these include genes involved in iron acquisition (*isd* gene cluster, *sirA*, and *fhuD2*) in line with the low iron availability within the host (40) (Fig. 4 and 5). These genes showed an overall higher expression level *in vivo* than at any of the *in vitro* growth phases (Fig. 5 and Table S6). Furthermore, genes of the type VII secretion system, three of the gene clusters encoding a family of conserved staphylococcal antigens (*csa2A*, -*3A*, and -*4A*), a number of genes encoding surface antigens, thermonuclease (*nuc*) and aureolysin (*aur*) genes, and genes encoding factors involved in immune evasion, such as the chemotaxis inhibitory protein-encoding gene (*chp*) and leukocidin genes (*lukA*, -*E*, and -*D*), were induced to higher levels than during *in vitro* growth. In particular, *lukE* and *lukD* were substantially more highly upregulated than their highest *in vitro* expression levels (Table S6). This indicated that these toxins could play important roles during staphylococcal infection and that they might provide interesting targets for development as vaccine candidates or as drug targets.

A second cluster of upregulated genes (Fig. 4, cluster VI) showed comparable expression levels to *in vitro* conditions, ranging from slight to intermediate induction, but in most cases, they did not reach the highest expression levels observed *in vitro*. Genes within this cluster belonged to genes associated with capsule biosynthesis (*capA* and *cap5H*), manganese transport (*mntA* and *mntH*), several toxins and proteases (*hlgABC*, *hld*, *eta*, *sspAB*, and *hla*), immune evasion factors (*sbi*, *spA*, and *scn*), and genes involved in oxidative stress response (*ahpC*), as well as several regulators of gene expression (*sarR*, *mgrA*, and *saeP*). Samples extracted from kidney and heart showed a differential expression profile for some of the genes falling into this cluster as well as genes in cluster III, and for these, expression in the heart was generally lower during earlier stages of infection and only increased toward the last time point.

Interestingly, we observed genes in cluster II that, despite being highly upregulated *in vitro* toward stationary phase, were downregulated or repressed *in vivo*. Genes within this cluster included the reporter gene for the σ^B^ regulon (*asp23*), the immunodominant staphylococcal antigen B-encoding gene (*isaB*), several surface-attached antigens (*sasD* and *ebpS*), and clumping factor A (*clfA*), as well as several important regulators of staphylococcal virulence gene expression (*sarA*, *sarZ*, *sarS*, and *agrA*). This suggests that the σ^B^ regulon might be repressed *in vivo*, and that the σ^B^ stress response is not important in regulating gene expression in infected organs. These data therefore suggest profound difference in bacterial virulence factor regulation between the *in vitro*-grown cultures and during host infection.

Overall, we found that several antigens known to be under investigation as vaccine candidates (34–37) (Fig. 5) were induced during infection, suggesting that these are actively expressed and could provide viable targets for vaccine development. A notable exception to this was *clfA*, which was found to be repressed during mouse infection, indicating that its expression would be lower during infection than under *in vitro* conditions. Expression of *spA* remained constant relative to the inoculum, a condition with high levels of *spA* expression, suggesting that its expression was maintained *in vivo*.

### Detoxified LukED is a safe and stable vaccine antigen.

**FIG 6:**
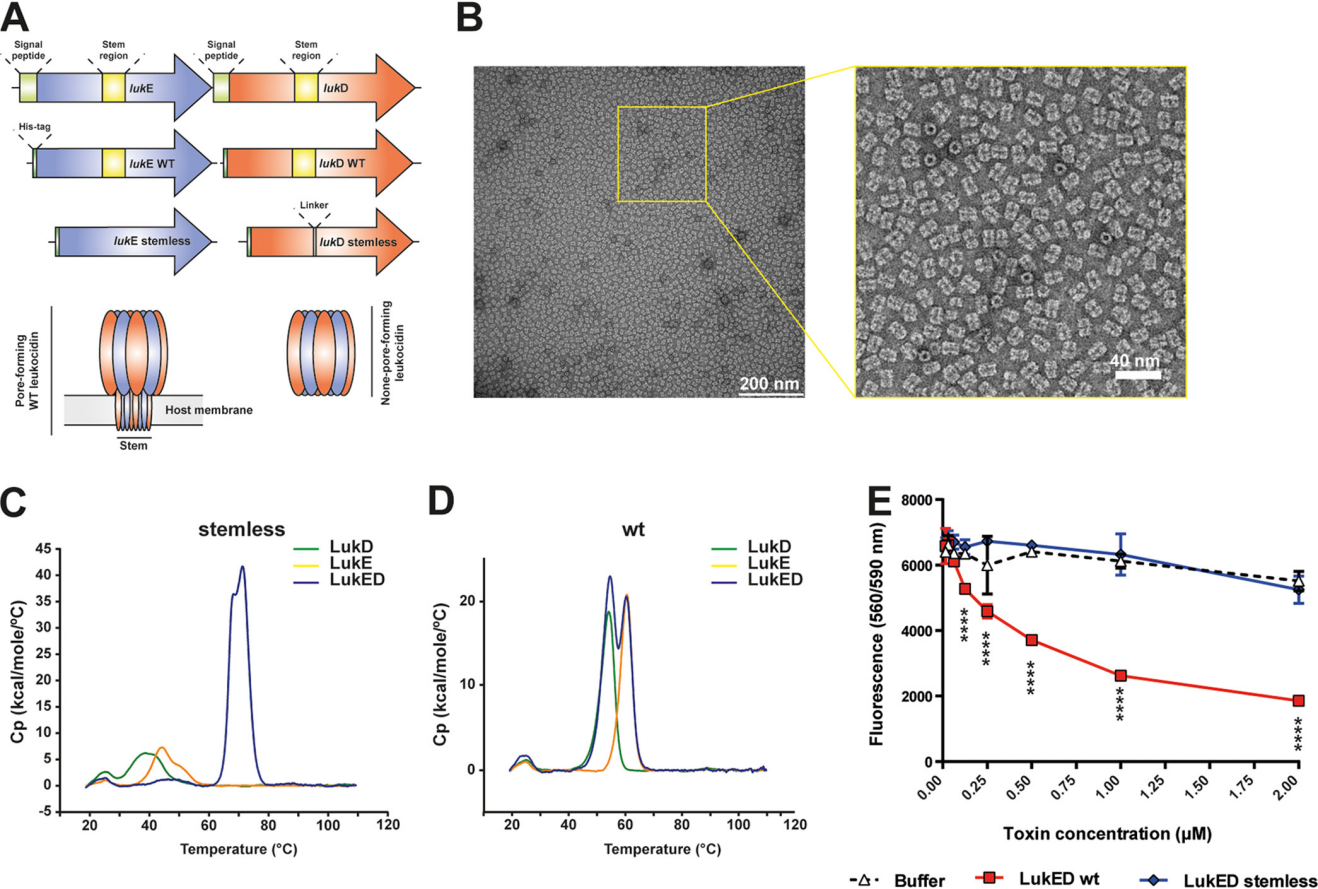
Characterization of the detoxified LukE and LukD proteins. (A) Schematic representation (from top to bottom) of the wild-type *lukED* locus for recombinant antigen production and single *lukE* and *lukD* expression constructs for wild-type protein expression as well as expression of stemless versions of LukE and LukD, respectively. Functional domains are indicated. At the bottom is a schematic representation of the oligomeric toxins formed in the wild-type cytotoxic LukED complex containing the pore-forming stem region and oligomeric engineered LukED lacking the stem region. (B) Negative-stain electron micrograph of spontaneously assembled detoxified LukED complex. Equimolar ratios of detoxified LukE and LukD were incubated at room temperature for 2 h prior to negative staining and EM preparation. (C and D) Differential scanning calorimetry of (C) stemless detoxified and (D) wt protein versions of LukD (green lines), LukE (yellow lines), and the LukED complex (blue lines). (E) Cytotoxicity assay for the indicated LukED versions. Cell viability was measured using a colorimetric assay as described in Materials and Methods. The colorimetric reaction was measured with a spectrophotometer at 560/590 nm, and the absorbance is directly proportional to the number of living cells in culture. Statistical analysis was performed using two-way ANOVA followed by Tukey's multiple-comparison test. Comparisons shown are between wt and detoxified LukED versions. Earlier time points with no indications showed no statistically significant differences. ****, *P* < 0.0001.

Having identified that the genes encoding the bicomponent toxin LukED were expressed at significantly higher levels *in vivo* than under any *in vitro* condition tested, we wanted to determine if these expression levels could serve as an indicator for their protective potential as vaccine antigens. LukED has been associated with dermonecrosis in a rabbit model (41) and also targets both human and murine cells (42), including T cells, monocytes, macrophages, DCs, neutrophils, erythrocytes, and endothelial cells (42–45). We therefore used structure-based design to engineer genetically detoxified versions of LukD and LukE to assess whether immunization with LukED can protect mice from developing dermonecrosis. To this end, we removed the stem region required for pore formation (Fig. 6A), and in the case of LukD, a short linker was found to increase the recombinant expression level in Escherichia coli. After expression and purification, the single proteins were mixed in an equimolar ratio to allow bicomponent complex formation. Negative-staining electron microscopy (EM) of the single proteins or the equimolar solution of the detoxified LukED antigens revealed that when both were present, the proteins autoassembled into a ring-like structure with an approximate radius of 8.5 nm (Fig. 6B). We performed differential scanning calorimetry (DSC) on both the detoxified stemless proteins and wild-type (wt) versions of LukE, LukD, and the respective complexes (LukED) (Fig. 6C and D, respectively). While the stemless versions of LukE and LukD were less thermostable than the wt proteins when assessed on their own, the stemless LukED complex was significantly more stable than the complex formed by wt LukED. *In vitro* cytotoxicity assays showed that our detoxified LukED did not induce cell killing, while treatment with wt versions of LukED resulted in a significant loss of cell viability (Fig. 6E). Therefore, the engineered LukED proteins formed highly stable complexes in solution which were nontoxic to eukaryotic cells.

### Immunization with the bicomponent leukocidin LukED protects mice.

**FIG 7:**
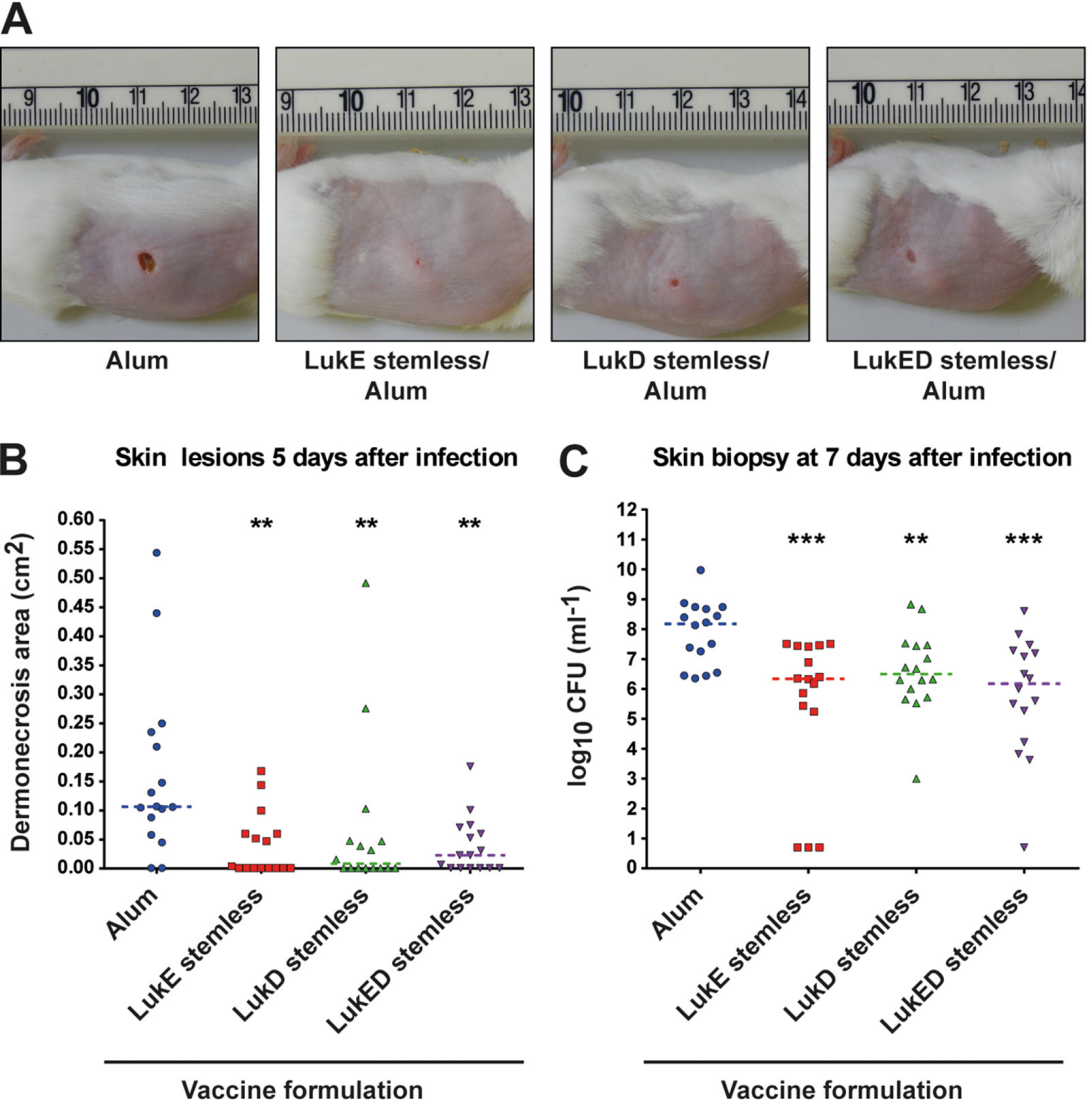
Detoxified LukED proteins elicit a protective immune response. Five-week-old, female CD1 mice were immunized twice intramuscularly with 10 μg the defined detoxified LukE or LukD or a combination of LukED proteins (see Materials and Methods for details). Ten days after the second immunization, mice were challenged subcutaneously with 1 × 10^7^ to 2 × 10^7^ CFU per mouse and (A) development of skin lesions of mice vaccinated as indicated was monitored. Representative photos of mice from the different groups at day 5 postinfection are shown. The mice selected for the figure were the ones that showed a dermonecrotic lesion size closest to the median value of the group. Median values for each single group were as follows: alum, 0.107 cm^2^; LukE stemless/alum, 0.004 cm^2^; LukD stemless/alum, 0.016 cm^2^; LukED stemless/alum, 0.023 cm^2^. (B) The area of skin lesion formed or (C) the number of bacteria recovered from skin biopsy specimens was determined after 5 or 7 days, respectively. The dotted line shows the median of the data, and statistical analysis was performed using a two-tailed Mann-Whitney test with *P* values indicated as follows: **, *P* < 0.01; ***, *P* < 0.001.

S. aureus causes substantial numbers of skin and soft tissue infections (SSTIs), and community-associated lineages such as USA300 have been particularly associated with these types of infections (46–50). As the *lukED* genes have recently been shown to be highly induced during *ex vivo* colonization of human skin explants and showed similar expression patterns to strain Newman when assessed *in vitro* (51), we selected the USA300 LAC strain to assess the ability of LukED immunization to prevent the formation of skin lesions in a mouse infection model. (46–51). We used the detoxified LukED antigens to immunize mice with either the single proteins or both proteins together, adjuvanted with alum. Ten days after the second immunization, mice were challenged with S. aureus strain USA300 LAC (2 × 10^7^ CFU per mouse) and the area of skin lesions and bacterial load of the infected wounds were determined at 5 and 7 days postinfection, respectively (Fig. 7B and C). Immunization with either the single or both engineered LukED proteins significantly reduced the area of skin lesions that developed (Fig. 7A and B), with large proportions of animals (12.5, 56.25, 56.25 and 43.75% of animals not showing lesions for alum, LukE stemless, LukD stemless, and LukED stemless immunizations, respectively) not developing any lesions at all (Fig. 7B). Moreover, ~100-fold less bacteria were recovered from vaccinated animals compared to the adjuvant control group (Fig. 7C). Therefore, vaccination with a detoxified form of the bicomponent toxin LukED, or a single monomeric component thereof, identified as highly upregulated *in vivo*, provided significant protection to animals infected with S. aureus compared to nonvaccinated controls.

## DISCUSSION

Selection of vaccine candidates is often driven by bioinformatics predictions and *in vitro* expression data of these virulence factors. While providing valuable information about regulatory networks that can affect their expression *in vitro* is vital for understanding pathogenicity, often *in vitro* conditions only poorly mimic the environment encountered during infections. Obtaining sufficiently comprehensive expression data from animal infection models or even from infected patients is challenging. The significant progress in high-throughput technologies for gene expression analysis provides an exciting opportunity for studying virulence factor expression levels at an individual strain resolution as well as within individual hosts after infection with pathogens. Such data can be crucial for understanding disease pathogenicity and for identifying and developing strategies for disease prevention and treatment.

To address this gap, in this study, we have developed an HT-qPCR assay allowing us to specifically and precisely monitor the expression levels of 84 genes involved in staphylococcal virulence during infection in individual organ tissues rather than in pooled samples containing between 25 and 250 pg (0.01 to 0.1% of total mixed RNA). In addition to providing overall expression trends for the virulence population, this enabled us to study variability of antigen expression levels within different host animals. This is particularly important when selecting promising vaccine antigens as these will ideally need to be expressed at high levels among all hosts.

Staphylococcal expression of virulence genes is intrinsically dependent on the growth phase of the culture with several major transcriptional regulators being activated at different growth phases and cell densities. By characterizing the gene expression profiles of 84 selected genes *in vitro*, it was not surprising that the majority of the genes were upregulated toward the stationary phase as many virulence factors such as toxins and superantigens are known to be, directly and/or indirectly controlled by the S. aureus quorum sensing system Agr, the σ^B^ stress response regulator, and the Fur iron-responsive regulator (52, 53). Our HT-qPCR assay has been recently used to compare the expression profiles of two groups of strains *in vitro*, and while overall similar trends of virulence gene patterns to strain Newman were observed, this analysis led to the identification of specific differences in the expression of distinct genes, namely, SpA and capsule biosynthetic genes, between the 2 groups of strains (54). This suggests that the HT-qPCR assay provides the opportunity to do a rapid virulence profiling of any given strain (circulating in, e.g., a hospital or the community).

In our mouse infection experiments, *agrA*-controlled virulence factors were significantly induced *in vivo* despite the complete downregulation of *agrA* expression. AgrA is the response regulator of the AgrAC two-component signaling system (9), and signal-dependent activation of AgrA, even when present at low protein concentrations, might account for the activation of its regulon (8). Agr regulation has been reported to be important for the development of acute disease, while low activity of Agr is associated with chronic staphylococcal infections, such as those involving biofilm formation (55). Expression of *agr* has been reported as completely absent under *ex vivo* bacteremia conditions (56), suggesting that it is either not expressed and/or not required or even detrimental to the bacterium during bloodstream infections. In our study, mice were infected intravenously, and therefore, bacteria were immediately exposed to the bloodstream before infecting the localized microenvironment and the high bacterial cell densities that may be formed in host kidney in the form of staphylococcal abscesses (57), where we did observe activation of the Agr-system.

Bacteria can modulate their gene expression using the alternative sigma factor B (σ^B^), which targets specific genes required for the adaptation and survival to environmental stresses, such as heat, high osmolarity, high ethanol concentration, high and low pH, and oxidizing agents (58). Surprisingly, we saw that *asp23* was completely repressed during infection, suggesting that the σ^B^ stress response is not important in regulating gene expression in infected organs. This was further supported by the repression of *clfA*, *sarA*, and *sarS* and the relatively low transcription levels of *fnbA*, *coa*, and *vwb*, all of which exhibited expression levels positively affected by σ^B^ (59). This is in contrast to *asp*23 expression, which was reported to be highly upregulated during Staphylococcus infection of human *ex vivo* skin explants (51), where σ^B^ regulation may play a more fundamental role. Our data agree with previous reports where σ^B^ appeared not to have a major contribution to progression of infection in a rat model of experimental endocarditis (60).

Unsurprisingly, there is evidence of upregulation of a large group of iron limitation-responsive genes. In line with our previous data (39), *fhuD2*, which encodes a staphylococcal lipoprotein involved in the uptake of iron hydroxamate, is upregulated around 10-fold *in vivo* and in the same cluster as other genes involved in host iron scavenging mechanisms, such as *sirA* and the Isd heme iron uptake system (61, 62). Expression of these genes is controlled by the ferric iron uptake regulator Fur, whose repression is relieved once iron concentrations are low, and the observed induction of these genes *in vivo* confirms the restricted availability of iron in the host (40).

One of the aims of this study was to identify genes that were upregulated *in vivo* and could significantly contribute to the ability of S. aureus to cause infection. Interestingly, we found that the bicomponent leukocidin *lukED* genes were some of the most highly induced genes during infection in kidneys (124- to 2,800-fold) and hearts (~19- to -612-fold) compared to the inoculum. Leukocidins are β-barrel bicomponent pore-forming toxins and can target numerous immune and nonimmune cells (63). Staphylococcal virulence correlates with the number of leukocidins that can be expressed by individual strains, and highly virulent strains have been found to express five different bicomponent leukocidins (64). A genome-wide association study recently established that pyomyositis, a severe infection of skeletal muscle caused by S. aureus and most commonly seen in children in the tropics, is critically dependent on a single toxin, the Panton-Valentine leukocidin (PVL) (65). While many leukocidins are only expressed in a subset of strains, LukED is present in up to 87% of staphylococcal strains and has activity against human, rabbit, and murine cells (64).

The massive upregulation observed here for *lukED* is in line with prior studies where LukED was identified as a major toxin involved in infection (42–45, 66). Regulation of the expression of leukocidins is complex and involves regulators such as Agr, SarA, Rot, SaeRS, and Fur (63). All known leukocidins are positively regulated by the SaeRS TCS, which responds to factors produced by neutrophils (67), and negatively regulated by Rot, which binds directly to the leukocidin promoters (68) and becomes deactivated on high cell density via the Agr system. Furthermore, the expression of some leukocidin genes, including *lukED*, has been shown to be linked to nutrient availability, in particular iron (44) and metabolites (69), whose availability may be restricted during infection. The significant upregulation of leukocidin expression during infection may be in response to any of several cues for the bacterium within the mouse kidney and heart tissue environment, including increased cell density, the presence of polymorphonuclear leukocytes (PMNs), and the lack of nourishment, ultimately resulting in the signal induction for upregulation of leukocidin expression. Previous studies have shown that the leukocidin genes were some of the most highly upregulated genes during incubation in human blood (70) and, more recently, in human skin explants (51). Beyond its role as a pore-forming toxin that targets a wide range of cells, including neutrophils and other leukocytes promoting virulence (42, 43, 66), LukED also enables S. aureus to acquire iron by lysing erythrocytes (44) and causes vascular leakage through targeting endothelial cells, which may be an important strategy for attracting nutrient-rich vascular fluid to the site of infection (45). All of the leukocidin genes monitored were significantly upregulated *in vivo*, including *hlgABC* and *lukAB*; however, *lukED* was induced to a significantly higher degree than the others throughout the complete time course of S. aureus infection (Table S6). Given that all these leukocidins are subject to the same Agr-Rot regulatory cascade, this might suggest that *lukED* can integrate an additional signal or signals during mouse infections, leading to increased activation or a different affinity for the known regulators leading to this increased expression (63).

As LukED leukocidins play and important role in causing disease by targeting primary human leukocytes critical for innate immune defenses and adaptive immunity, including neutrophils, monocytes, macrophages, dendritic cells, and effector memory T cells, as well as nonimmune host cells (42–44, 63, 71–74), they present an attractive target for the development of drugs and vaccines that can weaken staphylococcal virulence and disease severity. Impairing the ability of S. aureus to target the host immune system could reduce infection and promote pathogen clearance through the patient’s own immune response. We designed a recombinant form of the antigen lacking cytotoxicity by removing the stem portion responsible for forming the cytotoxic pore in both LukE and LukD proteins (66). The detoxified LukED complexes were substantially more thermostable than complexes formed with the wt proteins, which might increase their immunogenicity (75) and may also facilitate manufacturing and storage of these antigens. Critically, the detoxified LukED proteins conferred protection in a mouse model of staphylococcal skin infection either singly or in combination. This could be attributed to the mode of action of leukocidins in which the S-component leukocidin binds to the host cell membrane and subsequently recruits the F-component leukocidin to form the cytotoxic pore (64). Thus, elimination of only one of these leukocidins would be sufficient to prevent pore formation and would therefore confer protection. Leukocidins have been the targets for other antistaphylococcal interventions, including their use as promising therapeutic biologicals (76–80). Vaccination against LukED using toxoids has recently also been shown by Tam and colleagues to be effective in protecting mice against bloodstream infections (81). Interestingly, these toxoids had also previously been shown to inhibit the cytotoxic activity of their wt toxin counterparts as well as other leukocidins *in vitro* and reduced the bacterial burden in a murine bloodstream infection model (82). Here, we demonstrate that alternatively detoxified LukED proteins elicit a protective immune response in a mouse skin infection model and further corroborate the potential of LukED proteins as vaccine antigens. Both S. aureus Newman and USA300 express HlgAB, HlgAC, LukED, and LukAB, while USA300 also codes for the Panton-Valentine leukocidin LukFS-PV (63, 74, 83). A recent study has shown that LukED can act in conjunction with HlgABC to target mouse endothelial cells and facilitate host killing (45). Given the complexity of S. aureus factors involved in immune evasion and virulence, vaccine or drug formulations targeting only one factor are unlikely to result in full protection in a model of staphylococcal skin infection and the remaining leukocidins might partially compensate for the loss of LukED.

Therefore, the protective role of these leukocidins either previously reported for bloodstream infections or demonstrated here for skin infections supports the development of LukED, or fragments thereof, as part of a multicomponent vaccine. Recently multivalent formulations have been shown to result in protective immunity against S. aureus infections in a mouse model (84). The addition of leukocidins to the vaccine formulation could either improve the efficacy of these vaccines or allow tailoring of the vaccine formulation for invasive staphylococcal infections, where leukocidins play a crucial role in subverting host-immune defenses. The reduction in infection symptoms coupled with the reduced number of S. aureus recovered from skin wounds of immunized animals observed here further supports the development of LukED for vaccines against skin and soft tissue infections.

In summary, we have developed a highly sensitive and high-throughput qRT-PCR assay for the rapid quantification and profiling of staphylococcal virulence gene expression *in vitro* (54) and *in vivo*. Our study has highlighted the profound differences between the well-characterized gene expression observed *in vitro* and actual gene expression occurring during mouse infection. We have employed this assay to identify highly expressed virulence factors *in vivo* and used these data to design effective vaccine antigens. However, one of the hurdles of vaccine design for human-adapted pathogens such as S. aureus (85) is the lack of translatability to humans of preclinical data generated in animal models. Our assay could provide a feasible, high-throughput method that allows the determination of virulence gene expression patterns of samples directly from infected human patients as it requires only small quantities of bacterial RNA for successful, robust gene expression quantification from host-extracted samples. Therefore, it will be interesting to apply our assay to study gene expression profiles of virulence factors during infection of humans in an attempt to overcome the hurdle of species specificity the field faces.

## MATERIALS AND METHODS

### Bacterial strains and culture conditions.

The bacterial strains used in this study are listed in Table S2 in the supplemental material. S. aureus strains were grown at 37°C in tryptic soy broth (TSB) (Difco Laboratories) or in Trypticase soy agar (TSA) supplemented with 10 μg mL^−1^ chloramphenicol and 5% (vol/vol) sheep blood if required. For preparation of the bacterial challenge inoculum for infection studies in animals, an aliquot of bacteria (2 mL) frozen in phosphate-buffered saline (PBS) plus bovine serum albumin (BSA) (10% [wt/vol]) plus glutamate (10% [wt/vol]) was thawed, inoculated in 48 mL of TSB (starting from an optical density [OD] of 0.05) in flasks, and incubated at 37°C at 250 rpm until the OD_600_ reached 2. Bacteria were washed twice in equal volumes of PBS, collected by centrifugation for 10 min at 4,000 rpm, and suspended to 10^8^ CFU mL^−1^ to reach the necessary concentration for infection (10^7^ CFU per infectious dose). Escherichia coli strains were grown in Luria-Bertani broth (LB) or on LB agar plates. Antibiotic selection was used where appropriate.

### DNA manipulations.

General DNA manipulations were performed using standard procedures. The plasmid constructs used in this study (Table S4) were generated by cloning PCR products obtained with oligonucleotide primers listed in Table S5.

### Sample collection, RNA extraction, and cDNA synthesis.

We grew S. aureus strain Newman in TSB to the early-, mid-, and late-exponential, prestationary, and stationary phases and prepared mRNA for each time point. Bacterial mRNA samples for *in vitro* expression profiling were extracted from cells from TSA cultures grown to the indicated OD and stabilized with RNAprotect bacterial reagent (Qiagen) according to the manufacturer’s instructions. Bacteria were thereafter collected by centrifugation for 10 min at 3,200 × *g* and 4°C. Bacterial pellets were then either directly processed for RNA extraction or stored at −80°C. For samples from *in vivo* expression profiling, 8- to 10-week-old female CD1 mice (pathogen free) were infected intravenously with a sublethal dose of S. aureus (~1 × 10^7^ CFU per mouse) and organs harvested at defined time points. Samples for RNA extraction were stabilized using RNAprotect bacterial reagent (Qiagen, Germany) according to the manufacturer’s instructions. In the case of extracted host organs, the organs were collected in gentleMACS M tubes (Milteny Biotech) containing 2 to 4 mL of RNAprotect and immediately homogenized. Larger cell debris was removed from the homogenized samples by centrifugation at 100 × *g* for 5 min, and bacteria were thereafter collected by centrifugation for 10 min at 3,200 × *g*. Bacterial pellets were then either directly processed for RNA extraction or stored at −80°C. For RNA extraction, the bacterial pellet from either *in vitro* cultures or *in vivo* infections was resuspended in 1 mL of TRIzol reagent (Ambion) and lysed in a FastPrep-24 homogenizer (MP Biomedicals) using three cycles of 60 s at 6.5 m s^−1^, followed by 5 min of incubation in ice after each cycle. RNA was extracted from the suspension using Direct-zol RNA MiniPrep kit (Zymo Research), applying an on-column DNase digestion step using the RNase-free DNase kit (Qiagen) according to the manufacturer’s instructions. Residual DNA was removed by a second DNase treatment using RQ1 DNase (Promega), followed by RNA purification using the PureLink kit (Ambion) according to the manufacturer’s instructions. RNA quality was assessed by gel electrophoresis and an Agilent 2100 Bioanalyzer, and the absence of contaminating DNA was confirmed by qRT-PCR. cDNA was synthetized using the SuperScript first-strand synthesis system for RT-PCR (Invitrogen-Life Technologies) according to the manufacturer’s instructions, using a random hexamer primer for reverse transcription (RT) on 300 to 4000 ng of total RNA.

### Determination of bacterial RNA concentration in host-extracted RNA samples and qRT-PCR.

Mouse RNA was spiked with defined quantities of bacterial RNA and cDNA prepared as described above. qRT-PCR was performed using Platinum SYBR green qPCR SuperMix-UDG (Invitrogen-Life Technologies) using ROX as internal control on a STRATAGEN Mx3000P qPCR system using the following cycling parameters: 95°C for 10 min, followed by 45 cycles of 95°C for 30 s, 55°C for 30 s, and 72°C for 30 s, then 95°C for 1 min and 55°C for 30 s, and finally 95°C for 30 s. The amount of bacterial RNA in each sample was determined using 16S rRNA primers Sa_16s_+332_F and Sa_16s_+437_R (Table S5) (39) and relating each sample to a calibration curve.

### Design of TaqMan probes, preamplification of cDNA samples, and TaqMan qRT-PCR assays.

TaqMan qRT-PCR assays were designed on unique and conserved regions within the respective target genes of the following strains: Newman, FPR3757, Mu50, MW2, N315, and COL. Forward and reverse primers were designed to amplify fragments between 71 and 278 bp (Table S5). The indicated quantities of cDNA prepared as described above were used for preamplification using TaqMan PreAmp master mix (Invitrogen) according to the manufacturer’s instructions. In brief, assay mixtures to be tested were mixed to a working concentration of 180 nM for each assay (0.2×) and used for preamplification as per the manufacturer’s instructions with the following cycling parameters: 95°C for 10 min, followed by 14 to 20 cycles (as indicated for each experiment) of 95°C for 15 s and 60°C for 4 min. Preamplified samples were diluted 1:5 in TE buffer (10 mM Tris [pH 8.0], 0.1 mM EDTA) prior to loading onto the Fluidigm 48.48 Dynamic Array IFC chip. Chip loading and qPCR were performed exactly as in the manufacturer’s instructions using the following cycling parameters: 50°C for 2 min, followed by 95°C for 10 min, and then 40 cycles of 95°C for 15 s and 60°C for 1 min. Final data were analyzed using Genex, applying interpolate calibration using a control sample. Samples were normalized to the expression levels of *gyrB*, and relative expression values to the reference culture were calculated using the ΔΔ*C_T_* method.

### Data analysis.

Hierarchical clustering was performed using R-studio in R version 3.5 and heatmap3 with Euclidian distance and a complete linkage model. K-medoids-clustering analysis was performed in CLC Genomics Workbench using Euclidean distance on sample values while subtracting the mean gene expression levels, and the data were graphed using GraphPad Prism 6. Correlation analysis was performed in Biovinci 1.1.5, and correlation data were exported and graphed using R-studio in R version 3.5 and heatmap3.

### Expression and purification of LukED.

For the expression of recombinant noncytotoxic forms of the LukE and LukD proteins, the *lukE*_stem_pET and *lukD*_stem_pET plasmids were generated as follows: the complete *lukE* gene was amplified from S. aureus strain NCTC 8325 genomic DNA and cloned into the pET15TEV expression vector by polymerase incomplete primer extension (PIPE) cloning (86). The signal peptide was removed by whole-plasmid PCR using the primers LL_56_14 and LL_57_14, generating plasmid lukE_pET. The stem-loop region was removed using primers LL_08_14 and LL_09_14 and whole-plasmid PCR, generating plasmid lukE_stem_pET. The *lukD* gene lacking the signal peptide was amplified using primers LL_50_14 and LL_51_14 from NCTC 8325 genomic DNA and cloned into plasmid pET15TEV using PIPE cloning (86), obtaining the lukD_pET construct. The primers LL_04_14 and LL_05_14 were used to remove the stem region, through whole-plasmid PCR, generating plasmid lukD_stem_pET. As the expression of the LukD_stem protein resulted in low yield, the amino acid sequence PSGS was inserted as linker in the position previously occupied by the stem-loop devoid region. The primers LL_06_14 and LL_07_14 were used to generate plasmid lukD_PSGS_pET, using the lukD_stem_pET plasmid as the template.

The plasmids were transformed into the E. coli EnPresso B system (Sigma-Aldrich), and expression of the His-tagged proteins was induced according to the manufacturer’s instructions. The recombinant proteins were purified via Ni-nitrilotriacetic acid (NTA) affinity chromatography according to the manufacturer’s instructions, using CHAPS {3-[(cholamidopropyl)-dimethylammonio]-1-propanesulfonate} solubilization. The N-terminal His tag was cleaved on column using tobacco etch virus (TEV) protease, and purified protein was collected in the flowthrough. The buffer of the LukD Δstem-PSGS and LukE Δstem proteins was then exchanged by dialysis in 20 mM Bis-Tris–150 mM NaCl (pH 6.5) for further analyses.

### Differential scanning calorimetry.

The thermal stability of purified recombinant LukE and LukD protein variants was assessed by differential scanning calorimetry (DSC) using a MicroCal VP-Capillary DSC instrument (GE Healthcare) and standard operating conditions. Briefly, protein samples were prepared at a concentration of ~0.5 mg mL^−1^ in buffer containing 20 mM HEPES–300 mM NaCl (pH 7.4). The DSC temperature scan ranged from 10°C to 110°C, with a thermal ramping rate of 200°C per h and a 4-s filter period. Data were analyzed by subtraction of the reference data for a sample containing buffer only, using the Origin 7 software (MicroCal).

### Negative-staining electron microscopy.

Equimolar ratios of LukE and LukD stemless recombinant antigens at a final concentration of 0.6 mg mL^−1^ total protein were mixed in 20 mM Bis-Tris buffer (pH 6.5) supplemented with 150 mM NaCl and 1.0 mM CHAPS detergent and incubated together for 2 h at room temperature. Samples were added onto carbon-coated copper grids and stained with 1% uranyl acetate. Imaging was performed using an FEI Tecnai G2 Spirit microscope operating at 120 kV.

### Cytotoxicity assay.

Cells of the THP-1 cell line (ATCC TIB-202) were grown as single-cell suspensions in T75 cell culture flasks in RPMI supplemented with 10% of heat-inactivated fetal bovine serum (FBS) and passaged when the culture reached a concentration between 8 × 10^5^ and 1 × 10^6^ cells/mL. LukED wt, LukED tagless, and LukED stemless complexes were generated by mixing 20 μM each single component and incubating the mixture for 30 min at room temperature. To evaluate the viability of mammalian cells after exposure to S. aureus leukocidin complexes, THP-1 cells were plated in 96-well flat-bottom tissue culture treated plates at 1 × 10^5^ cells per well in RPMI supplemented with 10% heat-inactivated FBS. Cells were exposed to serial 2-fold dilutions (2 to 0.016 μM final concentration) of LukED wt, LukED tagless, and LukED stemless complexes for 1 h in 37°C with 5% CO_2_. After the exposure, CellTiter (Promega) was added to each well and the cells were incubated for an additional 4 h at 37°C in 5% CO_2_. The colorimetric reaction was measured with a spectrophotometer at 560/590 nm. The absorbance is directly proportional to the number of living cells in culture.

### Acute mouse infection model.

Eight- to 10-week-old female CD1 mice (pathogen free) were infected intravenously with 100 μL of a sublethal dose of the S. aureus Newman strain (~1 × 10^7^ CFU per mouse). To assess gene expression *in vivo*, infected hearts and kidneys were collected at 2, 4, 7, and 14 days. Each single organ was first homogenized in 2 to 4 mL RNAprotect, and RNA was extracted to determine gene expression. Organs from noninfected mice served to extract RNA as a control for background and unspecific amplification. To evaluate the presence of bacteria, the numbers of CFU per milliliter of organ homogenate were determined for each mouse organ by serial dilution of the respective homogenate in PBS and replica spotting on TSA plates.

### Mouse skin infection model.

Five-week-old female CD1 mice (pathogen free) were immunized two times (0 and 14 days) intramuscularly (50 μL for each leg) with 10 μg of each antigen (LukE and LukD-Δstem) adjuvanted with alum. Immunized mice were infected subcutaneously with a sublethal dose of S. aureus strain USA300 LAC (~1 × 10^7^ to 2 × 10^7^ CFU per mouse) 10 days after the second immunization. Five days after the infection, mice were anesthetized and photos of the skin lesions were taken with a Nikon Coolpix S9600 MP4 Land camera in order to measure the lesion area using the ImageJ NIH software (National Institutes of Health, USA). Mice were sacrificed 7 days after the infection, and the lesion areas were collected using an 8-mm AcuPunch biopsy punch (Acuderm, Inc., USA). Each skin sample was homogenized in 2 mL PBS, and serial dilutions of the homogenate were spotted in double (10 μL) on TSA plates to determine the CFU counts.

### Ethics statement.

All animal studies were carried out in compliance with current Italian legislation on the care and use of animals in experimentation (Legislative Decree 26/2014) and with the Animal Welfare Policy and Standards. Protocols were approved by the Italian Ministry of Health (authorizations 185/2011-B and 278/2015-PR). After infection, mice were monitored daily and euthanized when they exhibited defined humane endpoints preestablished in agreement with internal Animal Welfare Policies.

### Statistical analysis.

At least two independent experiments, run under the same conditions, were performed for all studies. Statistical analysis was performed using Graph Pad Prism 6. Expression data were reported as a logarithm to generate a Gaussian distribution, and outliers were determined using the ROUT method (*Q* = 1%). Statistical significance was determined by one-way analysis of variance (ANOVA) followed by Holm-Sidak’s test or two-way ANOVA followed Tukey’s multiple-comparison posttest. Significance values are expressed as follows: ns, not significant; *, *P* < 0.05; **, *P* < 0.01; ***, *P* < 0.001; ****, *P* < 0.0001.
