# Supplementary material for: In Vivo Gene Expression Profiling of Staphylococcus aureus during Infection Informs Design of Stemless Leukocidins LukE and -D as Detoxified Vaccine Candidates
Source: Microbiol Spectr. 2023 Jan 23;11(1):e02574-22. doi: 10.1128/spectrum.02574-22 (PMC9927290; doi:10.1128/spectrum.02574-22)
Supplement: Supplemental file 1 — Tables S1 to S5 and Fig. S1 to S5. Download spectrum.02574-22-s0001.pdf, PDF file, 1.9 MB [file spectrum.02574-22-s0001.pdf]

Supplementary Tables

Table S1 Taqman assays

Target gene	Forward Primer 5'-3'	Reverse Primer 5'-3'	Probe 5'-3'	Gene	Function
adsA	CATCACTTTCACCACGAATGTTTG	GCGTCTAACATTAAATCAGGCTTTTC	TTACATACAAATGATATCC	NWMN_0022	adenosine synthase A
agrA	CTCGCAACTGATAATCCTTATGAGG	GTAACGAAAATAATGTTACCAACTGGG	GATATTCAACTTTCAACTG	NWMN_1946	AgrA - accessory gene regulator A
ahpC	GAAATCTTACCATTTACAGCGCAAG	GCCTAATTTTTGTAATTCTTCATATTGG	CTATCCTGCTGACTTCTC	NWMN_0372	Alkyl hydroperoxide reductase subunit C
asp23	CAAGCATACGACAATCAAACTGGTG	GCAGCGATACCAGCAATTTTTTC	CGTCAAAAACAACAAGAAC	NWMN_2086	alkaline shock protein 23
atl	CGAAACAGCACCAACGGATTACTTA	CAGCATAGTTATTCATTGAACGTGCAA	ACTGCACCGACACCC	NWMN_0922	autolysin
aur	GAGCACTTTATCACCAGCAGCATTAG	GTTTTTACATCAGTAACAGCGTAATCTTG	GAGGTGACTCAAAAGAG	NWMN_2536	zinc metalloproteinase aureolysin
cap5H	GCTGAAAAACCAGTCCTCTAAAGAATC	CAAATCCAATATAACTGTATTCACCAATG	TAAGATTCATCGCTTGG	NWMN_0102	capsular polysaccharide 5
capA	CAACTTATCAACATCCAAGTTAAAAGTGG	TTTGGTGCGACTTTAACTGCTG	CCGAAGATTATGAGTG	NWMN_0095	capsular polysaccharide synthesis enzyme CapA
capA2	GCCTGACAAATATACTGCTTCTACTC	GACTGCAAACTACTTTGAACATTTTGG	GTCCTCAAGTGATTTAG	NWMN_2563	capsular polysaccharide biosynthesis protein capA2
chp	GGAATCAGTACACACCATCATTCAG	ATTTCTCAAACGTTCATCTAATTTTCC	CCGTTTCCTACAAATG	NWMN_1877	chemotaxis-inhibiting protein CHIPS
clfA	CAACGAATCAAGCTAATACACCG	GTTGTTGAAACATTTTCCGCATTTG	GTGAATCAAACAAGTAATG	NWMN_0756	clumping factor A, fibrinogen-binding protein A
clfB	GGATAGGCAATCATCAAGCACAAG	GCTATCTACATTCGCACTGTTTGTG	CAATATGATAGAAACACC	NWMN_2529	clumping factor B
coa	GAAATAAAACCACAAGGTACTGAATCAACG	GCTTCATATCCAAATGTTCCATCG	CAATTTAACAAAACACC	NWMN_0166	staphylocoagulase
csa1A	GAAGATATGGTAGCTAAAGGCATGGTTC	GTGCGGTTTTCCTTCATCC	CTACTATGTCGATGTGACT	NWMN_0042	staphylococcal tandem lipoprotein
csa1B	CTCAAATGGCAATTCAAAATAAAGG	CCTGAGCTACTTCGTATTTCTCCGTC	GGCATGCTTTTGAAGATAG	NWMN_0043	staphylococcal tandem lipoprotein

Target gene	Forward Primer 5'-3'	Reverse Primer 5'-3'	Probe 5'-3'	Gene	Function
csa1C	CAACAAAAGGATATTATTA TGTAAATGC	CCATATTGAACAAAGAACTT AAAGTTT	ACGGAAGACCCCAAGAG	NWMN_0044	staphylococcal tandem lipoprotein
csa1D	CTGAAATGGTTGTTCAAC CTAAAGG	CTTAGGTCTACCATCTTCAT CATGAAG	CTAAGACTACAACCGGG	NWMN_0045	staphylococcal tandem lipoprotein
csa2A	CGCTTTATGAAATTGATGG TCACG	CTTATCTACCTTTAACAACA TACGGTCTTC	CAGTTGTTGGATCAGAT G	NWMN_0148	staphylococcal tandem lipoprotein
csa3A	GAACCGCTAAAGGCCATT ATTTTGTTAC	CTGCTTGAGCTTATCATCTT TTACTTC	CCGGAATGGTAAACTAC	NWMN_0403	staphylococcal tandem lipoprotein
csa3B	CAAGGTGGAAGATCAAAA ACTTAAAG	GGAACATTACTATTAATTGA AACGTCGCC	GACAATATGCCGATTTC	NWMN_0404	staphylococcal tandem lipoprotein
csa3C	CAACCAAAAGGCGAGAGT TTAAAATC	CTATCCTATTATTCACCAAT TTCACTGG	GAGAACTGCTAAAGGAA G	NWMN_0405	staphylococcal tandem lipoprotein
csa3D	GTTTTATCGCAAGGATAAA TTACCTGATAG	CAATTCTTTAAGATTTGCAT ATTGACTG	GTTGAAATGAAAAATAAT	NWMN_0406	staphylococcal tandem lipoprotein
csa3E	GGCAATAAATCAGACGAA TTGGATG	GGTAATTTATCCTTTTCATA AAATTTCTTAAC	GAAATACTCGAACGGC	NWMN_0407	staphylococcal tandem lipoprotein
csa3F	CAGCCAAAGGGCAAAGTG ATG	CCTACCATTTTCACTTCGTA TTTTTCC	GGTTTCTTTGTTTTGAAA G	NWMN_0408	staphylococcal tandem lipoprotein
csa3G	GGGTTATTAGATCTGAAAT GACAACAG	CCTGACAAAATATTCACCAG TGC	CTAAAGGTATGGTCATA CG	NWMN_0409	staphylococcal tandem lipoprotein
csa3H	GGTGATAAAGGGATGTGG ACG	GGGAATTTGCCCTTATTATA GAATGTC	ACACGGACTGCAAAG	NWMN_0410	staphylococcal tandem lipoprotein
csa3I	CCAATTAAAAGGAGAAAAT CTGGAATC	GGAATCATTTTATTATTTTC CATTTTC	CAGAAATACTAGAACGG C	NWMN_0411	staphylococcal tandem lipoprotein
csa3J	CCAAAAAGGCAACAGGTT ATTATTTTG	GTTTAAGTTTTTTGTCTTCTA CATTATCC	GAGAAAAAATATCGTGT TG	NWMN_0412	staphylococcal tandem lipoprotein
csa4A	GAACCACAAAAGGGTATT ATTTTATAAGTG	CTTGTCATTCGGTAGTGGC TTCG	GTAACGGCAGACCAAAG	NWMN_2379	staphylococcal tandem lipoprotein
csa4B	GAATTTGATAAAAGAGATA AAGGGACTTGG	CCTTTACTATCTTCAGTTAT TTCGGTG	TGCTCTATATCAATCGC	NWMN_2384	staphylococcal tandem lipoprotein
csa4C	CTTGGATAATTCACTCTAA AATGACAATAG	GAGTATCCTTTTTTATCTTC CCATAATTC	GACCGCAATACCAGAAC	NWMN_2385	staphylococcal tandem lipoprotein
eap	CGAAAATAAAGCTAAAAG AAACTATCAAG	GCTTTCTTAGCATATTTTAA ATCTTGTTCAC	CATTTTCAAATAAACCTT G	NWMN_1872	MHC class II analog protein
ebpS	AAAGGTGCAGCGATCGGT	GCGGCAGAAGCACTTTTAC TTG	CTCCAGCCAAACCTG	NWMN_1389	elastin binding protein
esaA	GTTGCTGAGTCTGGTTTG AAAAATGG	GATATTTTCGATGGTGTTTT AGCGTC	CATGATTGTTATCCCAG	NWMN_0220	EssA, TVIISS

Target gene	Forward Primer 5’-3’	Reverse Primer 5’-3’	Probe 5’-3’	Gene	Function
esaB	CAGCACGTAAAAGTAACATTTGATTTTAC	TTTCAACAAGTAATTGACCTTTCGTC	ATAATTACGGCACATATG	NWMN_0221	EsaB, TVIISS
esaC	GCTGAACAATATAAGCGATTAGAATTTAATTTGAGTT	TTAACAGTTACACTGTCCTTATTAGCACTA	CCTTTGCTGTGCTTTC	NWMN_0224	EsaC
essA	CGACTCGCTTGAATGAAACTAAAAAAGTG	CCCCTACAGACATCAAAATGTACG	AAGACTTCGGAGAGTG	NWMN_0221	EssA, TVIISS
essB	GATTCCTAAGTCTTCAATTAAACCAGAAC	GGTGTATGATTGTCATTAATGTCATAATG	CCATATTTTATAGATGCTG	NWMN_0222	EssB, TVIISS
essC	TTCGCCAAGGATTATTCACCGTG	CTAATGGCGGTATAATGGAACG	CAATACAGAAGAACAATAC	NWMN_0223	EssC, TVIISS
esxA	GAGTCCAGAGGAAATCAGAGCAAAA	CCTTGTGCACGTGTTAAATCAGATAAAA	CTTGCCCGTAAGATTG	NWMN_0219	EsxA secreted protein
esxB	TCGCTGAGTATATCGAAGGTAGTGA	CGGTTGTACTAATTCTTCTTGAAACTTTGC	TTGGCGAACTGTCCTTC	NWMN_0225	EsxB secreted protein
eta	GCTTTCTTGATTTGGATTCACCTTTTATG	GCCAGACATGAAAAATGTTGTGAACAC	AATAATGTGAAAGAACAAT	NWMN_1082	exfoliative toxin A
fatB	CTATAAAATGCGTGGCGAGAAAAAAG	CTGATAAGCCCATTTCTTTCATTACATC	GTTAAAGAAACTGTTGAAG	NWMN_0705	ferrichrome ABC transporter lipoprotein
fbp	CACAAAATCAATCAACCTGATAATGAC	AATCTTGAAAAGTTTGGATGGATTGAC	AAAATAGACAAAACCATC	NWMN_1119	fibronectin/fibrinogen-binding protein
fhuD2	CCAACAGTAGTTGTTGACTATAATAAGCA	GCAGTTGTTTCTTCCCAATCTTTCTT	CTTTACCAACAATTTTCC	NWMN_2185	hydroxamate siderophore binding lipoprotein (FhuD2)
flipR	GCCAAGGTGATGTGAAGAAAGCAG	CGAGTCGATTTCACCGTTTTTAACAAC	GTCCTTTAGAAGAGAACAG	NWMN_1067	FLIPr
fnbA	ATTGAAACAATAGAAGAAACGGATTCATC	CTTCAAAGTCAATTGGATTTGATTCCTC	CCATACTGCTGTGGATAG	NWMN_2399	FnbA, C-term truncation in Newman
fnbB	GCGACATCAACTGAGCAACCATC	GAAGTTTCTACTTTTGGTGCTTGCACAG	TAACAACAGAAGAAGCAC	NWMN_2397	FnbB, C-term truncation in Newman
geh	CACATCAAATGCAGTCAGGAAAGC	CTTGTCGTTCAGAATCTTGCTTTACTTG	GTGGAACAGTGACAGAAG	NWMN_0262 (truncated?)	triacylglycerol lipase
gyrB	GGTGACTGCATTGTCAGATGTAAAC	CTGCTTCTAAACCTTCTAATACTTGTATTTG	CCCAGCACCATAATTA	NWMN_0004	DNA gyrase subunit B
hla	TATAGTCAGCTCAGTAACAACAACA	TGCATGCCATTTTCTTTATCATAAGTGAC	ATGCCGCAGATTCT	NWMN_1073	alpha-hemolysin precursor
hlb	GTAATATTCAATGAAGCATTTGATAATGG	GGATATTTACTTACAATCGCTACGCC	CTGAAGGTAGCTACTCATC	NWMN_1926 (truncated)	beta-hemolysin
hld	TTAAGGAAGGAGTGATTTCAATGG	TGAATTTGTTCACTGTGTCGATAATC	GATATCATTTCAACAATC	NWMN_2624	delta-hemolysin, RNAIII

Target gene	Forward Primer 5’-3’	Reverse Primer 5’-3’	Probe 5’-3’	Gene	Function
hlgA	GCAGAAAATAAGATAGAAGATATCGG	TCAAATTGAATGTTTTGAGTTATAGC	GTGCAGAAATCATCAAAAG	NWMN_2318	gamma-hemolysin component A
hlgB	GTTACAGAACAACATTAAGTCGCAACAC	TGTCTGCCAGCTAAGAAGAGTTCATTAC	GAATAATGGTTGGGGAC	NWMN_2320	gamma hemolysin, component B
hlgB2	GCTACTGGGAATATTAACTCAGGCTTTG	GTGCATAATCAACGACGTTTACTGAATC	GGGAGCTAAATACAATG	NWMN_2320	gamma hemolysin, component B
hlgC	GATACTGAAGACATCGGTAAAGGAA	AGAGCTAATGAATCCTTGCATCTTTAA	TTATCAAAAGGACAGAAG	NWMN_2319	gamma-hemolysin component C
hlgC2	GATAAAACAAGTAATAAATGGGGCGTGAC	GAATGGCCATCGCATAGCTTTAAC	GATATTAAAGATGCAAGG	NWMN_2319	gamma-hemolysin component C
hysA	GGAATGGATTTTGAAAATCAGGAC	GATGAATCAGTACTTTTAATGCCAGTTCC	CATATTTCATATTAAACG	NWMN_2106	hyaluronate lyase
icaB	GGATGGTCATCATATTGCAAATGCA	AATTCGCTTTTCTTACACGGTGATAATTT	CCAGAGCACTATTTTC	NWMN_2567	intercellular adhesion protein IcaB
isaA	CGTTGATCAAGCACACTTAGTTGACTTAG	GCTCCATGACCATGTAGTACCATTTGAAG	CTCCAATCAAAGATGGTG	NWMN_2469	immunodominant antigen A
isaB	GGCAAGGACTTGAAAAAAGAAAATGGT	CGACAACTCTATTATGATCAACGACAAAC	ACCGCTATCAGCTTCC	NWMN_2537	immunodominant antigen B
isdA	GCAGTTGAACCTGGATATAAGAGCTTA	TGCTTTTTCAAATTCCAAATGCGTAGT	TCGTGCCACAAATTAA	NWMN_1041	iron-regulated heme-iron binding protein IsdA
isdB	GGAGAAAATTTGAAGTTTATGAAGGTGACA	TGTTTTCGCTTTTTTATATGGCGCTAA	CAGTGCAGATAAATTC	NWMN_1040	iron-regulated heme-iron binding protein IsdB
isdC	TAATTATCATCATCGCGACATTCAG	CCATTTTTCTTAATGTACTTTGCCGG	CAATACCAATGACACGTC	NWMN_1042	iron-regulated cell surface protein, isdC
isdG	CGAGACATGGGATTGAAACATTAGAAG	GGGCTACTTTCATCTTCATTTTTACTTC	ACAGTTTGGAAATCAAA	NWMN_1047	cytoplasmic heme-iron binding protein, isdG
isdH	GTTGCATCGGTCATTGTCAGTAC	GTTGCATTATTATTTTGATTTTCCG	CAAGCAGCAGAAAATAC	NWMN_1624	haptoglobin-binding surface anchored protein
ltaA	CTGTAGCAATAACGTCTCTAGCATTTTC	GGTGATGCTGGAAACCAAATAAC	CAAAAATCGTTTTAACG	NWMN_0886	glycolipid permease LtaA
ltaS	CCGTAATAACGATTACCTTGAAGACG	TTTTTTGCCTTTAAAGAATAGGAACACAC	TGAATCCTTATAGTTTAG	NWMN_0687	glycerol phosphate lipoteichoic acid synthase
lukD	GAAAGTTACAGAACTACGATTGATAGAAAAACA	ATTATTCATAATTTTGTGCGCCTCAACA	CCCCAGCCAATTGA	NWMN_1718	leukocidin LukD
lukE	GATGTTGGTCAAACATTAGGATATAACATTG	ATTGTTTTAGAATAATTAAATGAGCCATTGCCA	CTGACTGGAAATTACC	NWMN_1719	leukocidin LukE
lukA	GCAGCAACGACTCAAGCAAATTC	GTTTCAGTTCGTTTTGTGATTTTACCG	GAACATGTTGATAAGTCTC	NWMN_1928	leukocidin/hemolysin toxin family S subunit

Target gene	Forward Primer 5’-3’	Reverse Primer 5’-3’	Probe 5’-3’	Gene	Function
mgrA	GCTCAAAGACAAGTTAAT CGCTACTACTC	GTGCTAATTCAGTTACGACT TTCTTGAC	CCCACAATTTCTTGTC	NWMN_0655	MgrA - MarR family regulatory protein
mntA	GTGTGGAACAAGTGATTT TATCAGG	CACTTAATTCTGAAATTTGT CGATGAC	GGATGGTTTAGACGACC	NWMN_0603	ABC transporter ATP-binding protein (mntA)
mntH	GGAAACTGGATAACATCA ATGCAAG	GTCGTGTCATTTGAGCTAA GTCCATAC	GGCTATACTTTGCTATTC	NWMN_0971	Mn2+/Fe2+ transporter NRAMP family protein
nuc	CCTGTACAACCATTTGGC AAAGAAGC	GCAAGTCCCTTTTCCACTAA TTCC	CGCTATGGTAGAACATT G	NWMN_1236	thermonuclease
NWMN_0677	GTGAAACTGTTGAAGGTA AAGCTG	ACCATTGCGATTTCTTTACC	CAAATCATCAAAAGGTC C	NWMN_0677	hypothetical protein
NWMN_0851	CAATTGCAGTAGATGGCA TTATGGC	CTTTCCAAGTAATCGTGTAA ACGGCAG	CCAAAAGATAGCCAATT A	NWMN_0851	putative surface protein
NWMN_1231	GGTGCTGGAAAGTCAACG TTAATTG	GAAACATCGTTTTTTGGAAC ATTATACTG	AATTCTGGTGAGATATTT G	NWMN_1231	ABC transporter (ATP-binding protein) homolog
sak	GAGGTAAGTGCATCAAGT TCATTCGAC	GTCCCAGGTTTAATAGGAA ACTCGAC	GATGGTAAATGTGACTG G	NWMN_1880	staphylokinase (phage)
sarA	GAGTTGTTATCAATGGTCA CTTATGCTG	CTTTGTTTTCGCTGATGTAT GTCAATAC	GAATTTTCAATTAGCTTT G	NWMN_0588	SarA transcriptional regulator
sarR	GTCAACGCAACATTTCAA GTTAAGAAG	CTCTGAGCACTTAGCAATCT CTTTAGATG	TCAATTTGAACTATGAAG	NWMN_2195	SarR transcriptional regulator
sarS	GATGAGCGTAATACTTAC ATTTCAATATCTG	CTATCTTTTGGTATCATCTG TGATTCAC	CAGAACGTGTTACATTG	NWMN_0056	SarS transcriptional regulator
sarZ	GGTTACATTGTTTTAATGG CGATTG	CATCTTTCTCTTCACGTGTT CGAAC	CTTAGATTCTGGAACAC TG	NWMN_2286	SarZ - MarR family regulatory protein
sasA	GCGACAAATTTACAACAA GTACAATTTGG	CGATTGTCACGACTTGATCA ACATTTC	CTGCTGTTACACAAGTG	NWMN_2553	serine-threonine rich antigen
sasB	CTACTATGCAAACGAATA GTAAGCAAGG	GTAATTCTTGAAGCATCAGC AACTGC	GAATTAGCAACTGTAAAT G	NWMN_2061	methicillin resistance determinant FmtB protein, sasB
sasC	GGTTCAGGAGGACATCTA ACTTTAAAGG	TGCCGCACGTCTACTTCTCT TTTTC	GAGCTAGTTGCAATTGC	NWMN_1649	similar to FmtB protein, cell wall anchored, SasC
sasD	CCTTATGGCGGAGTAGTA CCACAAG	GCGTCGCATCATACAATTTC ATATTATAG	GCACAATATACTGAATTA G	NWMN_0078	similar to functionally unknown protein, SasD
sasF	CATTGATTGATCAATCACA AGATAAGTCG	CGATTTGATAATCCTTTATT CGTCC	TTACAAACGAAATTAGG	NWMN_2545	similar to functionally unknown protein, SasF
sasG	CGAGAAAATACCGCAAGG TCATAAA	TGTCTGGATTCTTGATTCCT GGTTT	CAGATCAAACGGAAAAA GTA	NWMN_2392	cell wall surface anchor family protein SasG
sbi	GAAGAACAACGTAACCAA TACATCAAAAC	GTAAAAAGCGTTTTGTTGTG CAACAC	GAAGTATTCTCTGAATCA C	NWMN_2317	immunoglobulin G-binding protein Sbi

Target gene	Forward Primer 5’-3’	Reverse Primer 5’-3’	Probe 5’-3’	Gene	Function
scn	CTTGCCAACATCGAATGAATATCAAAAC	GTCTTTTGACTTAAGAGCATACATTGC	GATGAACTAAATGTTAATG	NWMN_1876	staphylococcal complement inhibitor SCIN (phage)
scpA	CAGAGCAGTATATGCAGTACGTTCATGC	GATATTCTATTAAACGCCCAACTAAATC	TACCACAATCAACATCAG	NWMN_1403	(scpA staphopain)
sdrC	ATGAATAATAAAAAGACAGCAACAAATAGA	AGCAGTACCTACAGAATACTTTCTTATCGA	AAAGGCATGATACCAAATCG	NWMN_0523	Ser-Asp rich fibrinogen/bone sialoprotein-binding protein SdrC
sirA	CATGGACACAAAAACCGAAATTC	CATTTCTAACTTTTGACGCGACAATTAAG	CTAAGATTGTAGGTCAAG	NWMN_0059	siderophore compound ABC transporter binding protein, sirA
spA	CAAACCTGGTCAAGAACTTGTTGTTG	GCTAATGATAATCCACCAAATACAGTTG	CATGCAGATGCTAAC	NWMN_0055	Immunoglobulin G binding protein A, spa
sspA	CTTATATTCAAGTTGAAGCACCTACTGG	CTTTTAAAGCATGAGGATCACCGTG	GGTGTAGTTGTAGGTAAAG	NWMN_0918	V8 protease, glutamyl endopeptidase precursor
sspB	CAACAACAATTTGCTGGTTATGCTAAAG	TAAACAATTTTACCGTCTTTTATAACTGG	GTAATGCAAAAACTGG	NWMN_0917	cysteine protease, staphylopain
vWbp	GAAAAATAAATTGCTAGTTTTATCATTGG	TCCCCAGAAACCACTGCAC	GTGTATCACAAATTTGG	NWMN_0757	secreted von Willebrand factor-binding protein

Table S2. Strains used in this study

Strains	Relevant characteristics	Source or reference
***S. aureus* strains**		
Newman	MSSA, clumping factor overproducer, GenBank accession number NC_009641, UK, CC8, ST254, CP5	(1, 2)
USA300-FPR3757	GenBank accession number CP000255.1, USA, CC8, ST8, CP5 but does not produce capsule due to frameshift in *cap5D*	(3)
USA100 Mu50	GenBank accession number BA000017.4, Japan, CC5, CP8	(4)
USA400 MW2	GenBank accession number BA000033.2, USA, CC1, CP8	(5)
USA100 N315	GenBank accession number BA000018.3, Japan, CC5, CP8	(4)
COL	GenBank accession number CP000046.1, UK, CC8, CP5	(6)
***E. coli* strains**		
BL21 (DE3)	F− *dcm ompT hsdS* $_B$(r_B^- m_B^-) *gal* (λDE3)	Novagen

Table S3. *In vitro* expression clusters.

Gene	Locus tag	Function	Cluster
ahpC	NWMN_0372	Alkyl hydroperoxide reductase subunit C	1a
asp23	NWMN_2086	alkaline shock protein 23	1a
atl	NWMN_0922	autolysin	1a
cap5H	NWMN_0102	capsular polysaccharide 5	1a
capA	NWMN_0095	capsular polysaccharide synthesis enzyme CapA	1a
capA2	NWMN_2563	capsular polysaccharide biosynthesis protein capA2	1a
clfA	NWMN_0756	clumping factor A, fibrinogen-binding protein A	1a
csa1A	NWMN_0042	staphylococcal tandem lipoprotein	1a
csa2A	NWMN_0148	staphylococcal tandem lipoprotein	1a
csa4A	NWMN_2379	staphylococcal tandem lipoprotein	1a
eap	NWMN_1872	MHC class II analog protein	1a
ebpS	NWMN_1389	elastin binding protein	1a
fbp	NWMN_1119	fibronectin/fibrinogen-binding protein	1a
flipR	NWMN_1067	FLIPr	1a
hlgA	NWMN_2318	gamma-hemolysin component A	1a
hlgB2	NWMN_2320	gamma hemolysin, component B	1a
hlgC	NWMN_2319	gamma-hemolysin component C	1a
hlgC2	NWMN_2319	gamma-hemolysin component C	1a
icaB	NWMN_2567	intercellular adhesion protein IcaB	1a
isaB	NWMN_2537	immunodominant antigen B	1a
isdH	NWMN_1624	haptoglobin-binding surface anchored protein	1a
ltaA	NWMN_0886	glycolipid permease LtaA	1a
ltaS	NWMN_0687	glycerol phosphate lipoteichoic acid synthase	1a
lukD	NWMN_1718	leukocidin LukD	1a
lukE	NWMN_1719	leukocidin LukE	1a
mgrA	NWMN_0655	MgrA - MarR family regulatory protein	1a
NWMN_0851	NWMN_0851	putative surface protein	1a
NWMN_1231	NWMN_1231	ABC transporter (ATP-binding protein) homolog	1a
saeP	NWMN_0677	SaeRS auxiliary protein	1a
sarA	NWMN_0588	SarA transcriptional regulator	1a
sarR	NWMN_2195	SarR transcriptional regulator	1a
sarS	NWMN_0056	SarS transcriptional regulator	1a
sarZ	NWMN_2286	SarZ - MarR family regulatory protein	1a
sasB	NWMN_2061	methicillin resistance determinant FmtB protein, sasB	1a

Gene	Locus tag	Function	Cluster
sasF	NWMN_2545	similar to functionally unknown protein, sasF	1a
sasG	NWMN_2392	cell wall surface anchor family protein sasG	1a
scn	NWMN_1876	staphylococcal complement inhibitor SCIN (phage-encoded)	1a
scpA	NWMN_1403	Staphopain	1a
aur	NWMN_2536	Zinc metalloproteinase aureolysin	1b
mntH	NWMN_0971	Mn2+/Fe2+ transporter NRAMP family protein	1b
agrA	NWMN_1946	AgrA - accessory gene regulator A	1c
csa3A	NWMN_0403	Staphylococcal tandem lipoprotein	1c
esxA	NWMN_0219	EsxA secreted protein	1c
eta	NWMN_1082	Exfoliative toxin A	1c
geh	NWMN_0262	Triacylglycerol lipase	1c
hla	NWMN_1073	Alpha-hemolysin precursor	1c
hld	NWMN_2624	Delta-hemolysin, RNAIII	1c
isaA	NWMN_2469	Immunodominant antigen A	1c
mntA	NWMN_0603	ABC transporter ATP-binding protein (mntA)	1c
NWMN_0677	NWMN_0677	Hypothetical protein	1c
sasA	NWMN_2553	Serine-threonine rich antigen	1c
sasD	NWMN_0078	Similar to functionally unknown protein, sasD	1c
sdrC	NWMN_0523	Ser-Asp rich fibrinogen/bone sialoprotein-binding protein SdrC	1c
sspA	NWMN_0918	V8 protease, glutamyl endopeptidase precursor	1c
sspB	NWMN_0917	Cysteine protease, staphylopain	1c
adsA	NWMN_0022	Adenosine synthase A	1d
esaA	NWMN_0220	EssA, TVIISS	1d
esaB	NWMN_0221	EsaB, TVIISS	1d
esaC	NWMN_0224	EsaC	1d
essA	NWMN_0221	EssA, TVIISS	1d
essB	NWMN_0222	EssB, TVIISS	1d
essC	NWMN_0223	EssC, TVIISS	1d
esxB	NWMN_0225	EsxB secreted protein	1d
isdA	NWMN_1041	Iron-regulated heme-iron binding protein IsdA	1d
lukA	NWMN_1928	Leukocidin/hemolysin toxin family S subunit	1d
sbi	NWMN_2317	Immunoglobulin G-binding protein Sbi	1d
isdC	NWMN_1042	Iron-regulated cell surface protein, isdC	2a
isdG	NWMN_1047	Cytoplasmic heme-iron binding protein, isdG	2a
chp	NWMN_1877	Chemotaxis-inhibiting protein CHIPS	2b
isdB	NWMN_1040	Iron-regulated heme-iron binding protein IsdB	2b

Gene	Locus tag	Function	Cluster
sirA	NWMN_0059	Siderophore compound ABC transporter binding protein, SirA	2b
fatB	NWMN_0705	Ferrichrome ABC transporter lipoprotein	3
hlb	NWMN_1926	Beta-hemolysin (interrupted by phage)	3
hysA	NWMN_2106	Hyaluronate lyase	3
nuc	NWMN_1236	Thermonuclease	3
sak	NWMN_1880	Staphylokinase (phage-encoded)	3
sasC	NWMN_1649	Similar to FmtB protein, cell wall anchored, SasC	3
clfB	NWMN_2529	Clumping factor B	4
coa	NWMN_0166	Staphylocoagulase	4
fhuD2	NWMN_2185	Hydroxamate siderophore binding lipoprotein, FhuD2	4
fnbA	NWMN_2399	FnbA, C-term truncation in Newman	4
fnbB	NWMN_2397	FnbB, C-term truncation in Newman	4
vWbp	NWMN_0757	Secreted von Willebrand factor-binding protein	4
spA	NWMN_0055	Immunoglobulin G binding protein A, SpA	5

Table S4. Plasmids used in this study.

Plasmid name	Description	Source or reference
pET15TEV	*E. coli* expression vector, TEV protease site, Amp^R	(7)
lukE_pET	*lukE* missing the signaling peptide cloned into pET15TEV	This study
lukE_stem_pET	*lukE* missing the signaling peptide and stem-loop cloned into pET15TEV	This study
lukD_pET	*lukD* missing the signaling peptide cloned into pET15TEV	This study
lukD_stem_pET	*lukD* missing the signaling peptide and stem-loop cloned into pET15TEV	This study
lukD_PSGS_pET	*lukD* missing the signaling peptide and stem-loop with PSGS linker peptide substituting loop region cloned into pET15TEV	This study

Table S5. Primers used in this study.

Primer name	Sequence (5'-3')	Plasmid
**LL_56_14**	TCCAGGGCAATACTAATATTGAAAATATTGGTGATGGTG-	lukE_pET
**LL_57_14**	TTAGTATTGCCCTGGAAGTACAGGTTTTCG	
**LL_08_14**	AAATAGAAAGTTATGTCAGTGAAGTAGACAAGCAAAAC	lukE_stem_pET
**LL_09_14**	ACATAACTTTCTATTTTGTTTTTAGGAAGGTAATTGATTAAG	
**LL_50_14**	CTGTACTTCCAGGGCGCTCAACATATCACACCTGTAAGC	lukD_pET
**LL_51_14**	ATTAAGTCGCGTTATACTCCAGGATTAGTTTCTTTAGAATCCG	
**LL_04_14**	AAAATGAAGAAAGTTACAGAACTACGATTGATAGAAAAAC	lukD_stem_pET
**LL_05_14**	TAACTTTCTTCATTTTGATTTTTAGGTGCATAGTC	
**LL_06_14**	GAACCGTCTGGCTCTGAAAGTTACAGAACTACGATTGATAGAAAAAC	lukD_PSGS_pET
**LL_07_14**	TTCAGAGCCAGACGGTTCATTTTGATTTTTAGGTGCATAGTC	
**Sa_16s_+332_F**	GAGACACGGTCCAGACTCCT	16s-qRT-PCR
**Sa_16s_+437_R**	ACGATCCGAAGACCTTCATC	

Supplementary Figures

Figure S1

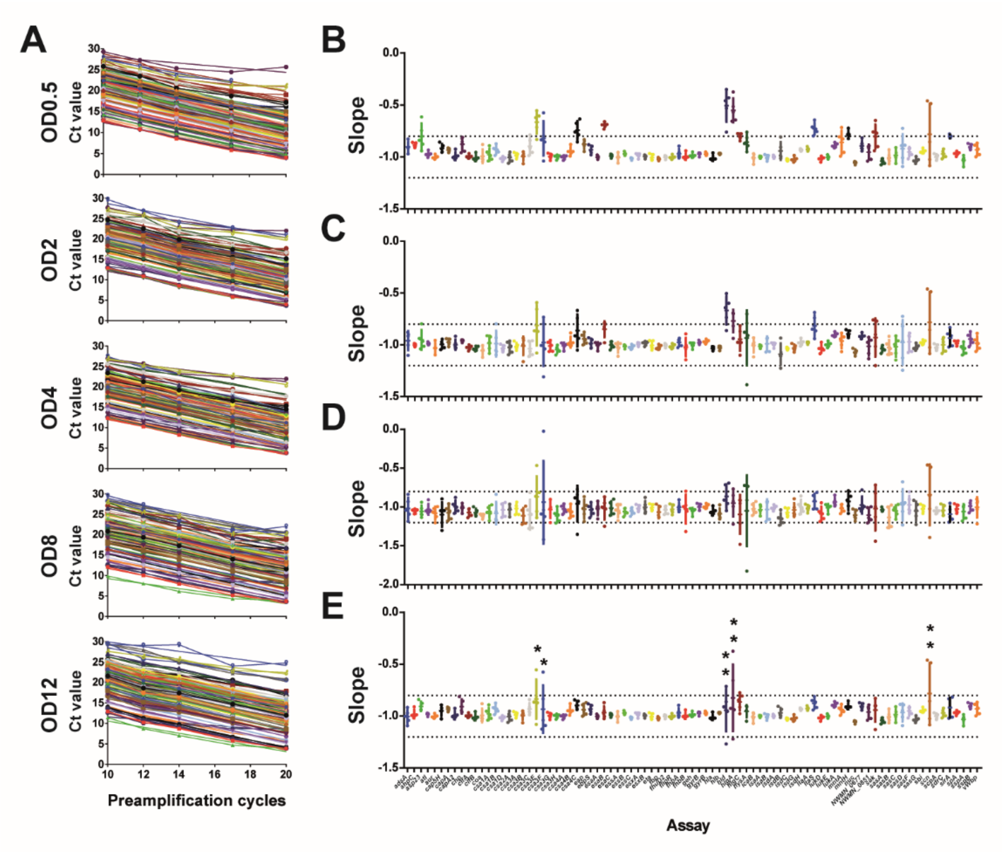

Figure S1 Linearity of individual target gene assays during preamplification. (A) The linear behavior of each target gene during preamplification was tested. Linear segments of each amplification curve were determined, and amplification slopes calculated. Depending on the individual assay, slopes began to deviate from -1 with preamplification cycles between 17 and 20 cycles for some assays. The majority of the assays performed well throughout all conditions tested. **(B-E)** Data curation. **(B)** Slopes before manual data curation considering all C_T values (10-20 preamplification cycles). Slopes considering only C_T values from **(C)** 10-17, **(D)** 10-14 or **(E)** C_T values of all preamplification cycles in the linear amplification range for each individual assay. A slope of -1 shows perfect linearity of the assay. Assays within a margin of 20% around the ideal slope (dotted lines) were considered functional. Some assays were functional only in a limited range of preamplification cycles. * non-functional assays were excluded, ** assays were redesigned and re-evaluated. If an assay performed in a linear fashion throughout both steps of the experiment and with 100% efficiency, we can assume that the resulting expression values should result in a slope of -1 when plotting the relevant C_T values against the number of pre-amplification cycles.

Figure S2

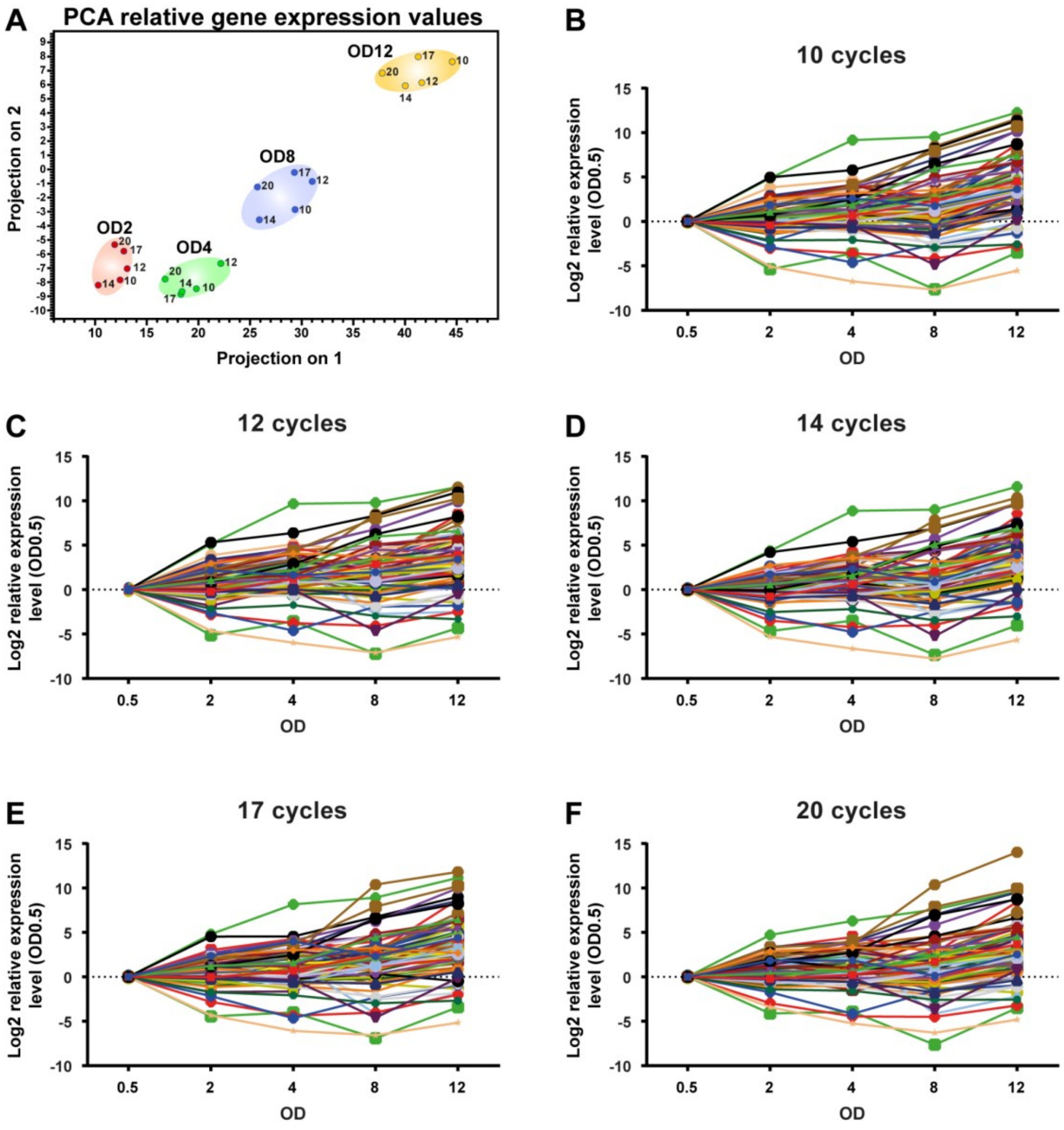

Figure S2 Assay performance. Relative expression levels for each gene within each preamplification set relative to exponentially grown cultures. Gene expression levels relative to OD 0.5 were calculated within each set of preamplification data and the impact of each preamplification cycles on overall data distribution and variability assessed using **(A)** principal component analysis and **(B)** analyzing individual gene expression profiles for each assay.

Figure S3

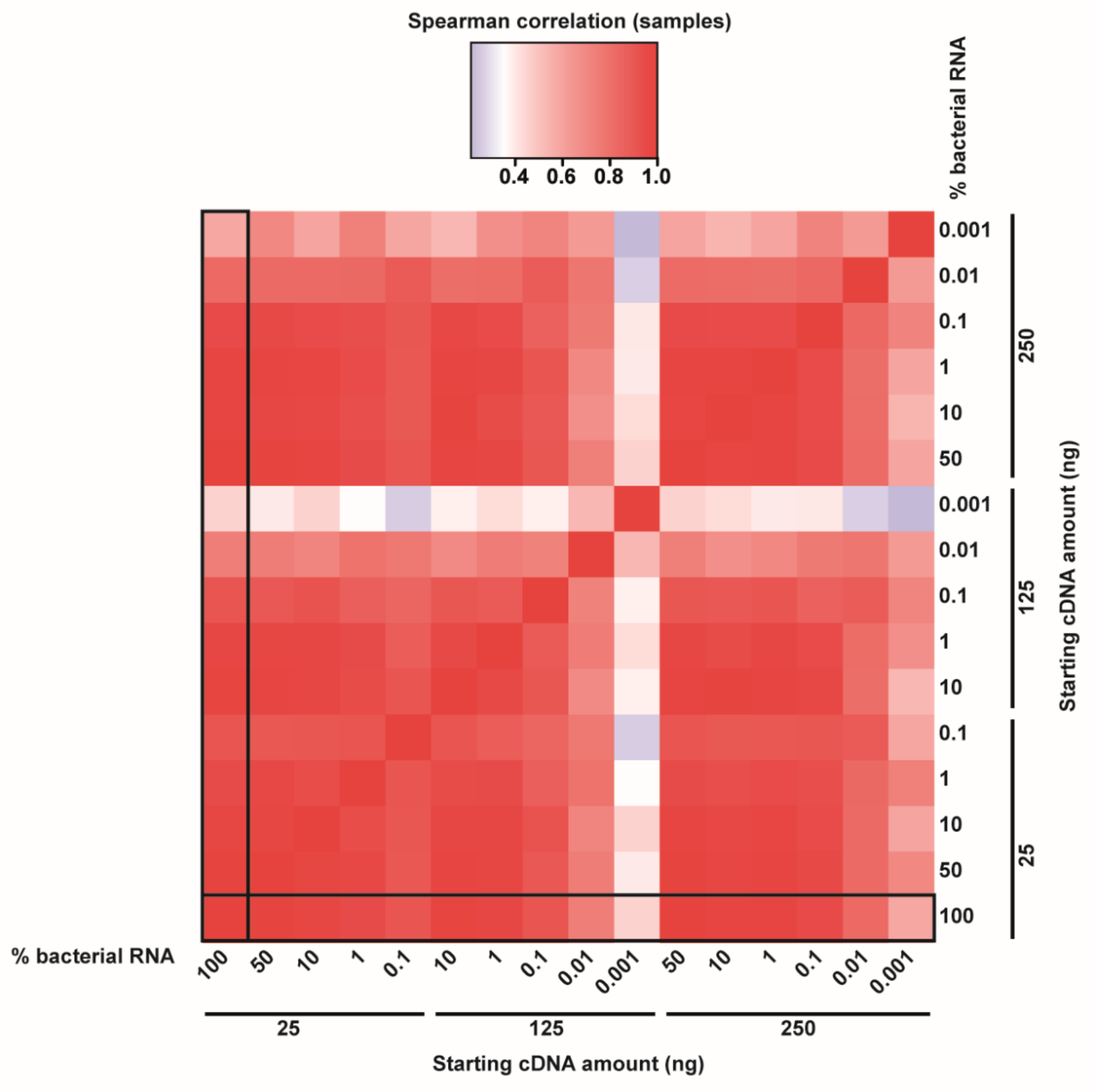

Figure S3 Analysis of correlation. Mixed samples of host and bacterial RNA (OD0.5 or OD12) were retrotranscribed and either 25, 125 or 250 ng of cDNA were used for pre-amplification (14 cycles). Relative gene expression values within each pair were calculated and reproducibility of gene expression values observed were assessed by calculating the Spearman's correlation coefficient. Increased starting cDNA concentrations greatly improved the detection limit and allowed reliable quantification down to 0.01/0.001% of bacterial RNA in the initial sample.

Figure S4

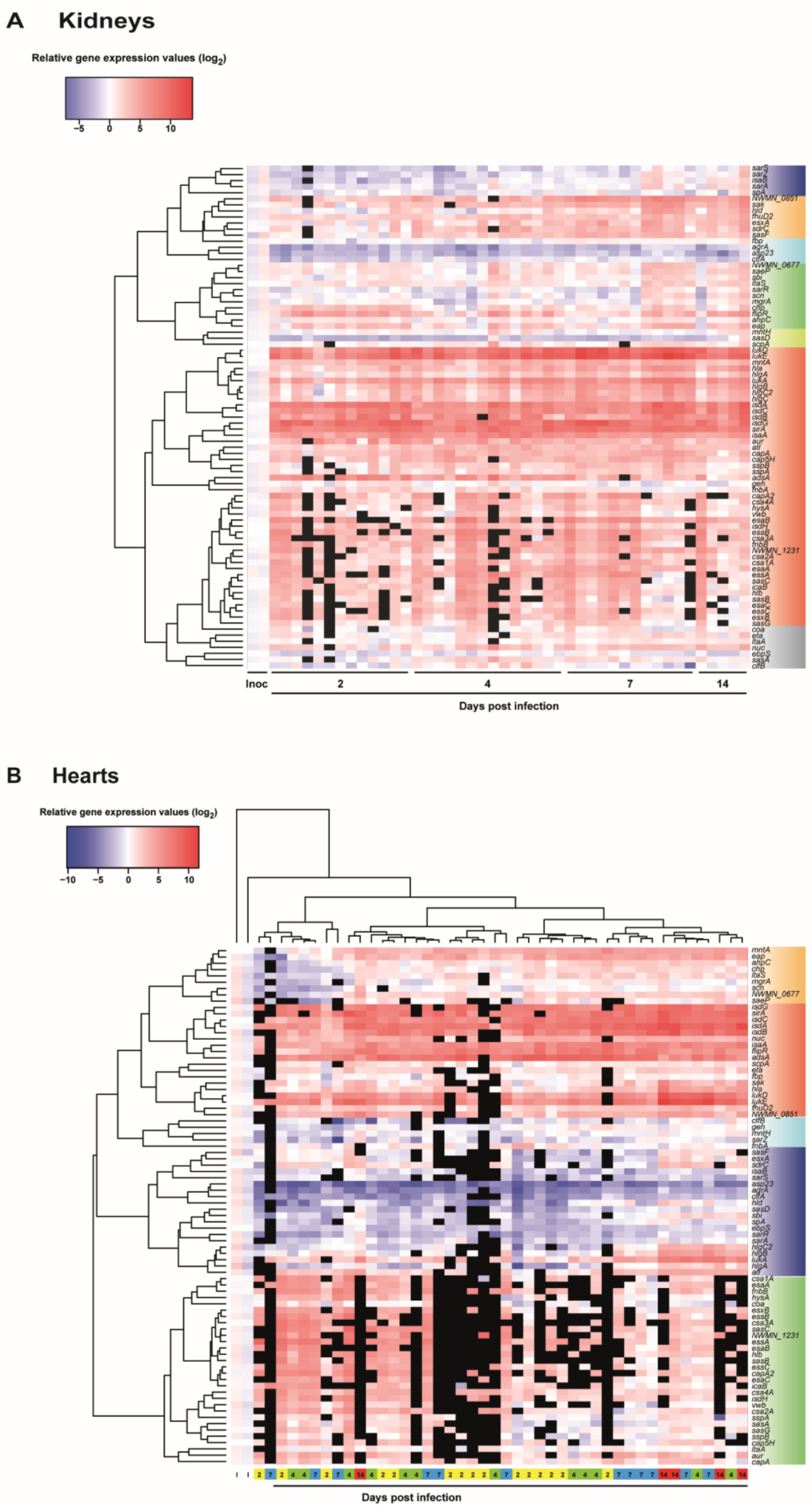
A Kidneys
Relative gene expression values (log2)
−5 0 5 10
Inoc
2
4
7
14
Days post infection
B Hearts
Relative gene expression values (log2)
−10 −5 0 5 10
Days post infection

Figure S4 *In vivo* expression levels of virulence factor genes in kidney tissue(A) and heart tissue (B). Heatmap of virulence factor expression profiles throughout infection of mouse kidneys and hearts. Expression levels were normalized to *gyrB* and are presented relative to the bacterial inoculum used for infection (OD 2 in TSB), black squares indicate missing data. Each column represents an individual mouse sample each row a Taqman gene assay.

Figure S5

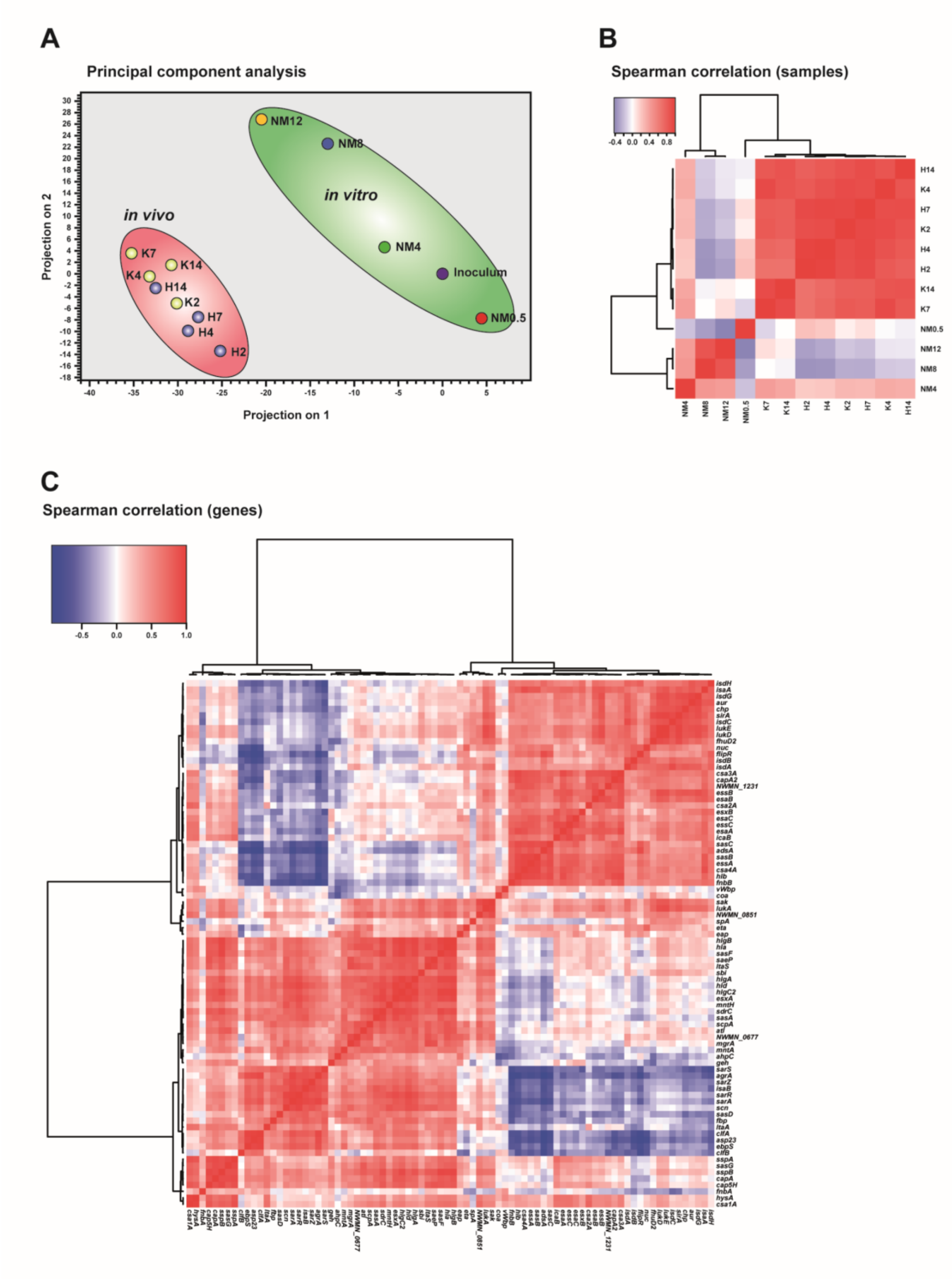

Figure S5 Correlation analysis of *in vitro* and *in vivo* samples. (A) Principal component analysis and **(B)** Spearman correlation coefficient analysis shows clear separation of *in vitro* and *in vivo* samples. (None of the *in vitro* conditions are truly reflecting *in vivo* gene expression levels). **(C)** Correlation of gene expression behavior identifies different co- and invers regulation patterns.

Supplementary References

1. Duthie ES, Lorenz LL. 1952. Staphylococcal coagulase; mode of action and antigenicity. J Gen Microbiol 6:95-107.
2. Baba T, Bae T, Schneewind O, Takeuchi F, Hiramatsu K. 2008. Genome sequence of *Staphylococcus aureus* strain Newman and comparative analysis of staphylococcal genomes: polymorphism and evolution of two major pathogenicity islands. J Bacteriol 190:300-10.
3. Diep BA, Gill SR, Chang RF, Phan TH, Chen JH, Davidson MG, Lin F, Lin J, Carleton HA, Mongodin EF, Sensabaugh GF, Perdreau-Remington F. 2006. Complete genome sequence of USA300, an epidemic clone of community-acquired meticillin-resistant *Staphylococcus aureus*. Lancet 367:731-9.
4. Kuroda M, Ohta T, Uchiyama I, Baba T, Yuzawa H, Kobayashi I, Cui L, Oguchi A, Aoki K, Nagai Y, Lian J, Ito T, Kanamori M, Matsumaru H, Maruyama A, Murakami H, Hosoyama A, Mizutani-Ui Y, Takahashi NK, Sawano T, Inoue R, Kaito C, Sekimizu K, Hirakawa H, Kuhara S, Goto S, Yabuzaki J, Kanehisa M, Yamashita A, Oshima K, Furuya K, Yoshino C, Shiba T, Hattori M, Ogasawara N, Hayashi H, Hiramatsu K. 2001. Whole genome sequencing of meticillin-resistant *Staphylococcus aureus*. Lancet 357:1225-40.
5. Baba T, Takeuchi F, Kuroda M, Yuzawa H, Aoki K, Oguchi A, Nagai Y, Iwama N, Asano K, Naimi T, Kuroda H, Cui L, Yamamoto K, Hiramatsu K. 2002. Genome and virulence determinants of high virulence community-acquired MRSA. Lancet 359:1819-27.
6. Gill SR, Fouts DE, Archer GL, Mongodin EF, Deboy RT, Ravel J, Paulsen IT, Kolonay JF, Brinkac L, Beanan M, Dodson RJ, Daugherty SC, Madupu R, Angiuoli SV, Durkin AS, Haft DH, Vamathevan J, Khouri H, Utterback T, Lee C, Dimitrov G, Jiang L, Qin H, Weidman J, Tran K, Kang K, Hance IR, Nelson KE, Fraser CM. 2005. Insights on evolution of virulence and resistance from the complete genome analysis of an early methicillin-resistant *Staphylococcus aureus* strain and a biofilm-producing methicillin-resistant *Staphylococcus epidermidis* strain. J Bacteriol 187:2426-38.
7. Cozzi R, Nuccitelli A, D'Onofrio M, Necchi F, Rosini R, Zerbini F, Biagini M, Norais N, Beier C, Telford JL, Grandi G, Assfalg M, Zacharias M, Maione D, Rinaudo CD. 2012. New insights into the role of the glutamic acid of the E-box motif in group B *Streptococcus pilus* 2a assembly. FASEB J 26:2008-18.
